# Structural Insight into IAPP‐Derived Amyloid Inhibitors and Their Mechanism of Action

**DOI:** 10.1002/anie.201914559

**Published:** 2020-01-28

**Authors:** Zheng Niu, Elke Prade, Eleni Malideli, Kathleen Hille, Alexander Jussupow, Yonatan G. Mideksa, Li‐Mei Yan, Chen Qian, Markus Fleisch, Ana C. Messias, Riddhiman Sarkar, Michael Sattler, Don C. Lamb, Matthias J. Feige, Carlo Camilloni, Aphrodite Kapurniotu, Bernd Reif

**Affiliations:** ^1^ Helmholtz-Zentrum München (HMGU) Deutsches Forschungszentrum für Gesundheit und Umwelt Institute of Structural Biology Ingolstädter Landstr. 1 85764 Neuherberg Germany; ^2^ Technische Universität München (TUM) Munich Center for Integrated Protein Science (CIPS-M) at the Department of Chemistry Lichtenbergstr. 4 85747 Garching Germany; ^3^ Technische Universität München (TUM) TUM School of Life Sciences Division of Peptide Biochemistry Emil-Erlenmeyer-Forum 5 85354 Freising Germany; ^4^ Technische Universität München (TUM) Institute for Advanced Study Lichtenbergstr. 2a 85748 Garching Germany; ^5^ Ludwig-Maximilians-Universität, Munich Department of Chemistry Center for Integrated Protein Science Munich (CIPSM) Nanosystems Initiative Munich (NIM) and Center for Nanoscience (CeNS) Butenandtstr. 5 81377 München Germany; ^6^ Università degli Studi di Milano Dipartimento di Bioscienze Via Giovanni Celoria 26 20133 Milano Italy

**Keywords:** amyloid formation, amyloid inhibitors, Aβ, solid-state NMR spectroscopy, peptides

## Abstract

Designed peptides derived from the islet amyloid polypeptide (IAPP) cross‐amyloid interaction surface with Aβ (termed interaction surface mimics or ISMs) have been shown to be highly potent inhibitors of Aβ amyloid self‐assembly. However, the molecular mechanism of their function is not well understood. Using solution‐state and solid‐state NMR spectroscopy in combination with ensemble‐averaged dynamics simulations and other biophysical methods including TEM, fluorescence spectroscopy and microscopy, and DLS, we characterize ISM structural preferences and interactions. We find that the ISM peptide R3‐GI is highly dynamic, can adopt a β‐like structure, and oligomerizes into colloid‐like assemblies in a process that is reminiscent of liquid–liquid phase separation (LLPS). Our results suggest that such assemblies yield multivalent surfaces for interactions with Aβ40. Sequestration of substrates into these colloid‐like structures provides a mechanistic basis for ISM function and the design of novel potent anti‐amyloid molecules.

## Graphical abstract

**ISM inhibitors** are highly dynamic assemblies and exchange between monomeric and high‐molecular‐weight states. In both states, the ISM peptide adopts a β‐loop‐like structure that provides a suitable surface for sequestration of Aβ40 in the colloidal state. ISM inhibitors thus exploit multivalency by self‐association to yield high substrate avidity.

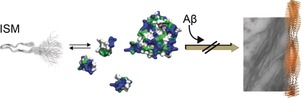

## Introduction

Peptides mimicking the cross‐amyloid interaction surface of the type 2 diabetes (T2D) human islet amyloid polypeptide (hIAPP) with the Alzheimer's disease β‐amyloid protein Aβ40/42, termed interaction surface mimics (ISMs), have been shown to be nanomolar inhibitors of amyloid self‐assembly of Aβ40/42.^1^ The molecular mechanism is, however, not well understood. The design of the ISMs was based on the finding that amyloids are generally composed of a β‐sheet‐turn‐β‐sheet structural motif and that IAPP uses the same two binding regions for both its amyloid self‐ and its cross‐amyloid hetero‐assembly with Aβ40/42.1a, 2 ISMs were thus derived by linking the two hot segments IAPP(8–18) and IAPP(22–28) in native or N‐methylated form to each other via different linkers, mostly tripeptide sequences consisting of identical amino acids; notably, these two segments are highly homologous to segments of the amyloid core of Aβ.^3^ High Aβ40/42 anti‐amyloidogenic activity was found for seven out of the 16 studied ISMs with six of them containing bulky hydrophobic/aromatic residues (e.g. LLL, III, FFF) in the linker tripeptide and one of them, termed R3‐GI, the RRR tripeptide.

It has been recognized recently that liquid–liquid phase separation (LLPS) plays an important role for self‐organization of membrane‐less cellular organelles.^4^ In particular, proteins containing low‐complexity sequences can form protein‐rich droplets.^5^ Phase separation is the driving force for the formation of membrane‐less cellular organelles such as nucleoli, stress granules, P‐bodies, and other cellular compartments.^6^ Similar to stress granules, hydrophobic small molecules undergo LLPS, adopt colloidal structures in aqueous environment,^7^ and recruit amyloidogenic proteins into their core in which amyloids adopt an altered structure that prevents amyloid neurotoxicity.^8^ The elevated local concentration facilitates interactions with the amyloid.

Herein, we show that R3‐GI is highly dynamic, can adopt a β‐like structure, and oligomerizes into colloid‐like assemblies. Our results suggest that formation of such ISM assemblies provides a multivalent surface for interactions with Aβ40, resulting in its sequestration into off‐pathway non‐toxic aggregates. The suggested mechanism provides a possible mechanistic scenario for the potent amyloid inhibitor function of ISMs.

## Results

**Figure 1:**
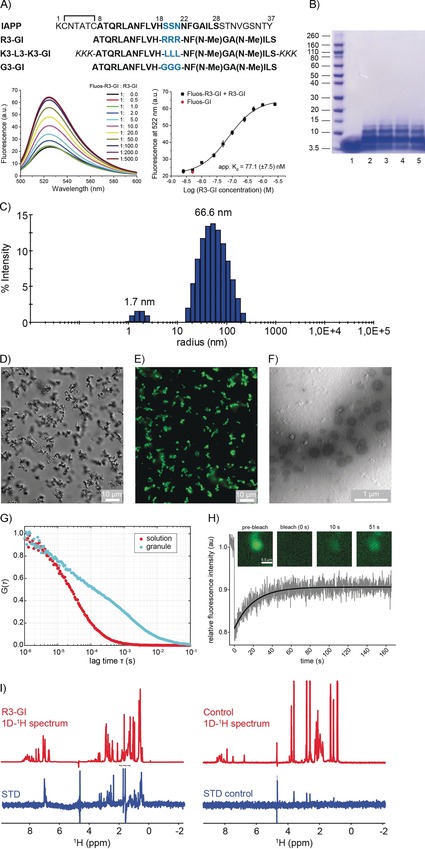
R3‐GI self‐assembly. A) Top: sequences and abbreviations of IAPP and the investigated ISMs. “Hot segments” of the IAPP interaction surface with Aβ40 are shown with black bold letters; residues in the IAPP loop and ISM linker regions are drawn in blue. The L3‐GI solubilizing tag “KKK” is shown in italics. Bottom: fluorescence emission spectra of N‐terminal fluorescein‐labeled R3‐GI (Fluos‐R3‐GI; 5 nm) alone and after titration with R3‐GI at the indicated molar ratios (pH 7.4). On the right, the binding curve is shown. The estimated app. *K*_d_ value for R3‐GI self‐association is 77.1(±7.5) nm. Data are means (±SEM) from three binding curves. B) R3‐GI oligomerization (500 μm) studied by cross‐linking with glutaraldehyde and NuPAGE. Lane 1: R3‐GI without cross‐linking; lanes 2–5: R3‐GI cross‐linked following incubation for 0 h (lane 2), 1 h (lane 3), 24 h (lane 4), and 48 h (lane 5). C) DLS studies of a 50 μm R3‐GI in 10 mm sodium phosphate buffer, pH 7.4 (containing 1 % v/v HFIP). D) Differential interference contrast (DIC) micrograph of R3‐GI (500 μm). The peptide was imaged immediately after dissolution into 10 mm sodium phosphate buffer, pH 7.4 (containing 1 % v/v HFIP) without filtration. Scale bar: 10 μm. E) Microscopy image of Fluos‐R3‐GI in fluorescence mode. F) TEM image of R3‐GI (500 μm) in 10 mm sodium phosphate buffer, pH 7.4 (containing 1 % v/v HFIP). Spherical assemblies were observed with diameters in the range of 200–400 nm. G) Fluorescence correlation spectroscopy (FCS) of a mixture of 454 μm R3‐GI and 454 nm Fluos‐R3‐GI showing that the translational diffusion inside the R3‐GI granules is strongly retarded. H) Fluorescence recovery after photobleaching (FRAP) of a R3‐GI granule. The fast recovery of the fluorescence intensity indicates that the majority of the peptide molecules are exchanging with the bulk solution within seconds. I) Saturation transfer difference (STD) NMR experiments of 1.25 mm R3‐GI in 10 mm sodium phosphate buffer, pH 7.4 (containing 1 % v/v HFIP and 10 % v/v D_2_O), showing that soluble, monomeric R3‐GI undergoes exchange with a high‐molecular‐weight oligomeric R3‐GI conformer. As a control, STD spectra of a 60 μm solution of the peptide GVAEPEQDCAVTSGE (Mr=1.492 kDa) are shown. The control peptide is monomeric under the conditions employed in the experiment.

Our studies focused on the two ISMs R3‐GI and K3‐L3‐K3‐GI for the following reasons (Figure 1 A and Table S1 in the Supporting Information): 1) R3‐GI was used in solution‐state NMR studies as the intrinsically low solubility of all ISMs with hydrophobic linkers makes them unsuitable for solution‐state NMR spectroscopy. 2) K3‐L3‐K3‐GI was used in MAS solid‐state NMR studies as it is a reasonably soluble and functional analogue (Figure S1) of the sparingly soluble but highly potent L3‐GI (LLL in the linker), which shows the largest effects in terms of substrate interaction.1b A third ISM, the non‐inhibitor G3‐GI containing the flexible GGG tripeptide as linker, was used as a control peptide.

### ISM Self‐Association and Exchange between Monomeric and Oligomeric States

Most of the ISMs self‐associate with apparent binding affinities (app. *K*_d_) in the low‐ to mid‐nanomolar concentration range.1b In the case of R3‐GI, an app. *K*_d_ value of 77 nm was determined by titrating synthetic N‐terminal fluorescently labeled peptide (Fluos‐R3‐GI) with R3‐GI (Figure 1 A). The high oligomerization propensity of R3‐GI was further confirmed by concentration‐dependent CD studies (Figure S2 D), chemical cross‐linking (Figure 1 B), and dynamic light scattering (DLS; Figure 1 C). In addition, differential interference contrast (DIC) microscopy, fluorescence microscopy, and transmission electron microscopy showed that R3‐GI aggregation resulted in granule‐like, high‐molecular‐weight structures (Figure 1 D–F). As expected, R3‐GI assemblies within these structures have a significantly retarded translational diffusion coefficient in comparison to particles in isotropic solution (Figure 1 G). Next, we recorded solution‐state NMR spectra of R3‐GI at a concentration of 1.5 mm (Figure S2 A). At this concentration, peptide self‐association should result in aggregates that are too large to be observable by solution‐state NMR spectroscopy. Unexpectedly, high‐intensity and high‐resolution spectra were obtained that are characteristic for monomeric, random‐coil peptides. The ^13^C chemical shifts, which are sensitive to secondary structure,^9^ yielded no indication for formation of α‐helical or β‐sheet secondary structure elements (Figure S2 C).

In order to resolve the apparent discrepancy between the NMR findings and the results of the other biophysical studies, we performed fluorescence recovery after photobleaching (FRAP) experiments (Figure 1 H). We found that the fluorescence of the granules recovered within seconds, indicating that peptides exchange between the peptide‐dense phase and bulk solution. In addition, we conducted saturation transfer difference (STD) experiments for R3‐GI (Figure 1 I). We observed very intense signals in this experiment, suggesting that R3‐GI undergoes chemical exchange between a monomeric random‐coil‐like conformation and an aggregated state that is too large to be observable by solution‐state NMR analysis. In contrast, a non‐aggregating monomeric peptide used as a control yielded no STD signals. Notably, similar behavior has been observed previously for the Alzheimer's disease Aβ peptide, which has been shown to exchange between a soluble and an aggregated state.^10^ These findings are in agreement with DOSY experiments (Figure S2 E, F). We observed a smaller apparent diffusion coefficient that is consistent with R3‐GI undergoing a transition between a monomeric and an aggregated state. Next, we quantified the amount of R3‐GI in the aggregated state versus solution. For this purpose, we determined the intensities of the fluorescent granules with respect to background (Figure S3). We found a partitioning coefficient of R3‐GI on the order of 2.9, which corresponds to an approximately threefold higher concentration in aggregates than in free solution.

### NMR Structural Characterization of R3‐GI

Subsequently, we recorded NOESY spectra to assign the resonances of R3‐GI (Figure S2 A) and to determine the structure of the peptide. N‐Methylation increases the population of a *cis* peptide conformer, and has been suggested to induce a turn structure similar to a proline.^11^ As expected, three sets of resonances are observed in the N‐methyl region (residues N15–L20). We estimated the populations of the three conformers G17(*trans*)–I19(*trans*), G17(*cis*)–I19(*trans*), and G17(*trans*)–I19(*cis*) to be on the order of 64 %, 32 %, and 4 % (Figure S4). The G17(*cis*)–I19(*cis*) conformer is not sufficiently populated to be observable by NMR spectroscopy. Furthermore, we found different sets of resonances at the N‐terminal half of the peptide (residues F8–H11; Figure S5), suggesting that N‐methylation assists in turn formation of the monomeric peptide.

**Figure 2:**
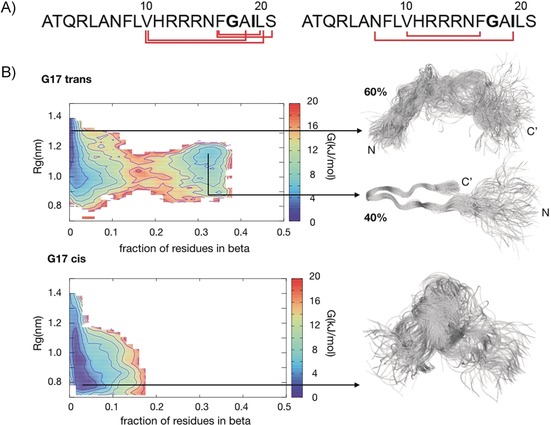
R3‐GI NOESY experimental data and molecular modeling of the monomer. A) Long‐distance NOE contacts plotted onto the R3‐GI peptide sequence for conformers 1 and 2. B) Free energy diagram and structural ensembles for R3‐GI.

The STD NMR and FRAP experiments demonstrate that R3‐GI exchanges between a monomeric and an oligomeric form. The experimental NOEs are thus transfer‐NOEs^12^ containing contributions from the monomeric and the oligomeric state of the peptide. In fact, the observed NOEs are very intense, underlining the exchange contribution to the NOEs. Figure 2 A summarizes the experimental long‐range ^1^H,^1^H NOE connectivities for R3‐GI. The observed contacts are indicative for a structure containing a loop. We investigated further the salt, temperature, and pH dependence for loop formation (Figures S6 and S7). Whereas the salt concentration did not have a significant impact on the intensity of the long‐range cross‐peaks in R3‐GI, we found that conditions of low pH significantly increased the intensity of the long‐range cross‐peaks. Similarly, we found that low temperatures increase the fraction of peptides adopting the turn‐like structure (Figure S7). Interestingly, the (N7–I19)^2^ cross‐peak intensity seems to correlate with the p*K*_a_ value of the histidine imidazole ring (Figure S8). We speculate that a lower pH and protonation of the histidine side chain is beneficial for loop formation in the aggregated state. At the same time, low pH has no influence on the population of the two conformers observed in the N‐terminal half of the peptide (Figure S4). We observed long‐range NOEs for both conformer 1 (G17(*trans*)–I19(*trans*)) and conformer 2 (G17(*cis*)–I19(*trans*); Figure 2 A). By contrast, the non‐inhibitor peptide G3‐GI shows only weak long‐range NOEs if any, suggesting that the loop‐like structure is not adopted for G3‐GI (Figure S9). These results are in good agreement with previous results and support the hypothesis underlying the design of the ISMs.1b

Conformational ensembles representing the R3‐GI conformers 1 and 2 were generated by metadynamic metainference^13^ using 221 and 35 inter‐residue distance restraints for the first and the second conformer, respectively (Table S4 and Table S5). Metadynamic metainference represents an extension of the inferential structure determination approach introduced by Nilges and co‐workers for heterogenous systems.^14^ Using this method, an optimal coupling of simulations and equilibrium experiments allows one to determine the overall ensembles of structures that are compatible with the experimental data, in this case with the NOE‐derived distances. The calculated ensembles for the two conformers are highly heterogeneous. In fact, a close inspection of the ensembles reveals significant differences. The G17(*trans*)–I19(*trans*) ensemble is characterized by an equilibrium between two populations. The first conformer is lacking any secondary structure and features a large radius of gyration (ca. 1.3 nm), while the second conformer is characterized by a loop forming a β‐like structure involving residues N7–V10 to S21. The free energy for members of the two different populations is rather similar, suggesting that conformers of the two populations may interconvert on a fast timescale (microseconds or less). By contrast, the ensemble for the G17(*cis*)–I19(*trans*) conformer does not show any indication for a loop‐like structure and is overall more compact with an average radius of gyration of 0.9 nm, reflecting the observed NOE between N7 and I19. The conformational ensembles suggest that the peptide is overall disordered in solution with some preference for a β‐like structure, in particular for the G17(*trans*)–I19(*trans*) conformer.

**Figure 3:**
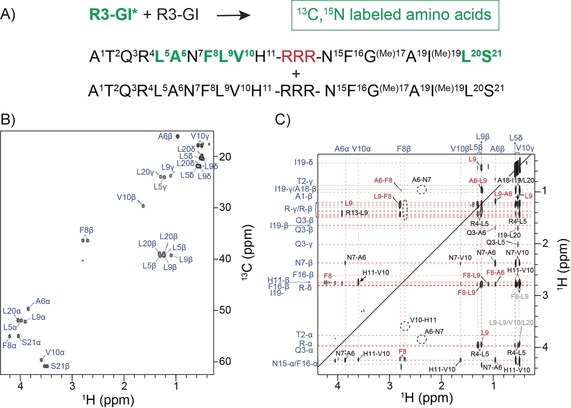
Identification of intermolecular NOEs for R3‐GI. A) Amino acid sequence and labeling scheme for R3‐GI. Residues labeled in green are uniformly enriched with ^13^C and ^15^N. For the NMR experiment, labeled and non‐labeled peptide was mixed in a 1:1 ratio. B) ^1^H,^13^C correlation spectrum of R3‐GI*. C) ^1^H (ω_1_),^1^H (ω_3_) correlation spectrum extracted from the 3D NOESY experiment. During ω_1_, protons were selected that are directly bonded to ^12^C, whereas ^13^C‐bound protons were filtered during ω_3_. In addition to trivial sequential connectivities (e.g., H11β–V10α) that appear only on one side of the diagonal, a number of symmetric cross‐peaks (in red) were observed that are due to intermolecular interactions.

The NOE intensities cannot easily be disentangled into contributions originating from the monomeric and the oligomeric state of the peptide. In order to probe peptide–peptide contacts in the oligomer, we prepared a mixed sample that contained 50 % unlabeled (R3‐GI) and 50 % labeled peptide (R3‐GI*; labeling scheme depicted in Figure 3). In the experiment, a magnetization filter element was applied during the first evolution period *t*_1_ to remove magnetization of protons that are directly bound to ^13^C nuclei, following a double half‐filter approach.^15^ After the NOESY mixing time, ^13^C‐bound protons were selected for detection. We found a number of sequential connectivities between labeled and non‐labeled residues within one peptide (e.g., H11β–V10α). These cross‐peaks are detected either above or below the diagonal, indicating that filtering of magnetization works in the intended way. In addition to these intramolecular sequential connectivities, we observed many correlations, which yielded a symmetric cross‐peak both below and above the diagonal (A6β–F8β, A6β–L9β, F8β–L9β, L9β–L9δ). These correlations are due to intermolecular connectivities as they involve potentially labeled amino acids in both evolution periods.

**Figure 4:**
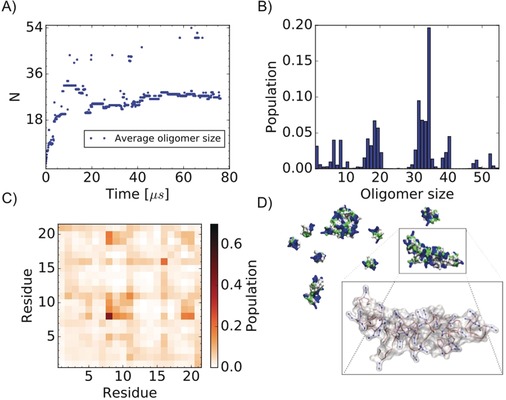
Coarse‐grain MD simulations of R3‐GI self‐assembly. A) Average oligomer size during the simulation. The simulation was performed using a total of 54 monomers. A value of *N*=54 would imply that a single oligomer is formed encompassing all of the 54 simulated molecules; by contrast, a value of *N*=1 would indicate that all 54 molecules are monomeric in solution and no oligomers are formed. B) Distribution of the oligomer size over the simulation. C) Intermolecular contact probability map averaged over the simulation. D) Surface representation for a transient oligomeric state (blue for positively charged, red for negatively charged, green for polar, and white for hydrophobic side chains). The Figure shows an ensemble of monomers and oligomers of different sizes, together with their surface properties. Arginine side chains (in blue) are solvent‐exposed. The inset shows a single oligomer enlarged, with arginine residues in ball‐and‐stick mode.

In order to gain more structural insight, we performed MARTINI coarse‐grain MD simulations (Figure 4).^16^ 54 monomers were solvated in a cubic box of 22.7 nm lateral length. The structure of the individual monomers was described by employing an elastic network centered around a representative G17(*trans*)–I19(*trans*) β‐turn‐like conformer. Interestingly, while the molecules self‐assembled quickly on the timescale of the simulation (after 10 μs, the average oligomer size was stable between 24 and 30 monomers), the oligomers were overall very dynamic, showing a broad distribution of sizes and a significant fraction of free monomers (Figure 4 A, B). Furthermore, monomers can exchange among oligomers on the microsecond timescale in agreement with STD experiments. The monomer–monomer interface is well defined and characterized by few intermolecular interactions (Figure 4 C). Arginine side chains are solvent‐exposed (Figure 4 D). Intermolecular interactions involve almost only amino acids 7–10. This is in agreement with the experimental intermolecular NOE contacts (Figure 3 C), suggesting that the dynamic oligomer may represent well the macroscopic behavior of R3‐GI at a small scale. As a control, five additional simulations were carried out to investigate the role of the linker sequence (Figure S11). First, the structure of the R3‐GI monomer was relaxed by removing the elastic network around the β‐like structure, which resulted in a more disordered peptide. Interestingly, the overall behavior of the system is robust with respect to this property. The resulting oligomer has similar dynamics and a similar intermolecular interface, while the average oligomer size is slightly decreased. Further control simulations with and without an elastic network were performed for G3‐GI and for the peptide (SG)_10_S with a supposedly totally flexible linker. For G3‐GI and (SG)_10_S, the oligomer dynamics disappears. Here, monomers self‐assemble rapidly into 54‐mers. Interestingly, differences can be observed for intermonomer interactions. While G3‐GI retains specific intermolecular interactions very similar to R3‐GI, the specific contacts are lost for the (SG)_10_S peptide. The simulations indicate that both R3‐GI and G3‐GI can form oligomers with robust intermolecular interactions. The three arginine residues in the loop of R3‐GI induce a β‐like structure that may determine the specific dynamic properties of the oligomers, which are essential for its inhibitory function.

### Substrate Interactions

**Figure 5:**
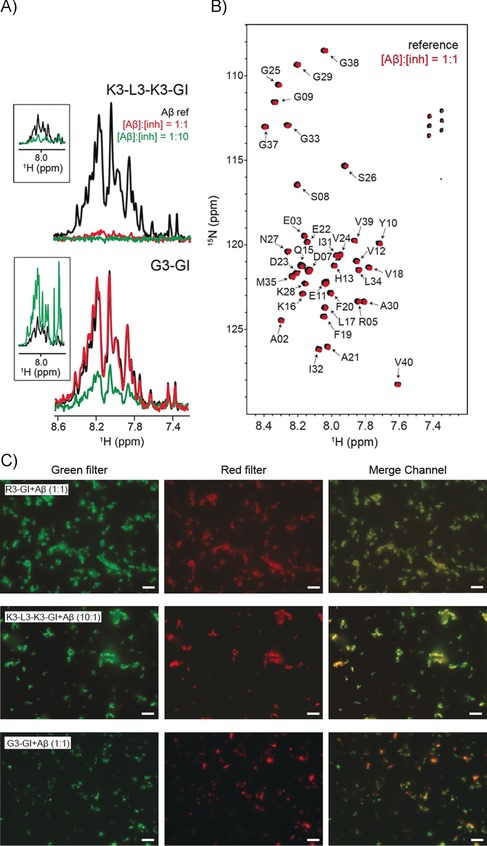
ISM–substrate interactions. A) ^15^N‐filtered 1D‐^1^H spectra of a 20 μm sample of ^15^N‐labeled Aβ40 in the absence (black) and presence of the ISM inhibitor K3‐L3‐K3‐GI (top), and the non‐inhibitor G3‐GI (bottom). At a molar ratio of 1:1, only 9 % of Aβ40 remains in solution after addition of K3‐L3‐K3‐GI, whereas the solubility of Aβ40 is almost unaffected upon addition of G3‐GI. At a tenfold excess of K3‐L3‐K3‐GI, the resonances of Aβ40 disappear quantitatively. At this molar excess, the intensities of Aβ40 are reduced only to ca. 28 % when G3‐GI is titrated to Aβ40. The inset shows the 1D‐^1^H spectra of the respective samples. At a molar ratio of 1:10 for Aβ/inhibitor, the spectrum is dominated by the resonances of the inhibitor. The disappearance of all resonances in the case of K3‐L3‐K3‐GI indicates that inhibitor and substrate co‐precipitate. B) 2D ^1^H‐^15^N HSQCs of Aβ40 incubated with K3‐L3‐K3‐GI (red) at a molar ratio of 1:1. The Aβ40 reference spectrum is represented in black. No Aβ40 chemical shift changes were observed after addition of the ISM inhibitor. C) Comparison of the DIC and fluorescence microscopy images of R3‐GI/Aβ40, K3‐L3‐K3‐GI/Aβ40, and G3‐GI/Aβ40. Whereas Fluor‐647‐Aβ40 incubated with either Fluos‐R3‐GI or Fluos‐K3‐L3‐K3‐GI yielded a perfect merge, Fluor‐647‐Aβ40 incubated with Fluos‐G3‐GI yielded a distinct spatial distribution of red and green fluorescent spots, suggesting that G3‐GI does not co‐localize with Aβ40. Scale: 10 μm.

To investigate substrate interactions, we turned to the ISM K3‐L3‐K3‐GI. Upon titration with Aβ40, hetero‐complexes precipitated quickly out of solution (Figure 5 A, top). As a control, monomeric Aβ40 was incubated with the non‐inhibitor G3‐GI, and no effects on Aβ40 solubility were observed (Figure 5 A, bottom). At the same time, the chemical shifts of Aβ40 upon titration of the ISM K3‐L3‐K3‐GI are not affected (Figure 5 B). At a tenfold molar excess of the ISM peptide K3‐L3‐K3‐GI with respect to Aβ40, the 1D–^1^H solution‐state NMR spectrum is almost empty (Figure 5 A, inset), indicating that the two peptides co‐precipitated. By contrast, high intensities were observed for the control sample Aβ40⋅G3‐GI (Figure 5 A, inset). Furthermore, fluorescence microscopy showed that K3‐L3‐K3‐GI or R3‐GI co‐localize with Aβ40 in the aggregates. By contrast, no co‐localization with Aβ40 was observed in the case of G3‐GI (Figure 5 C).

**Figure 6:**
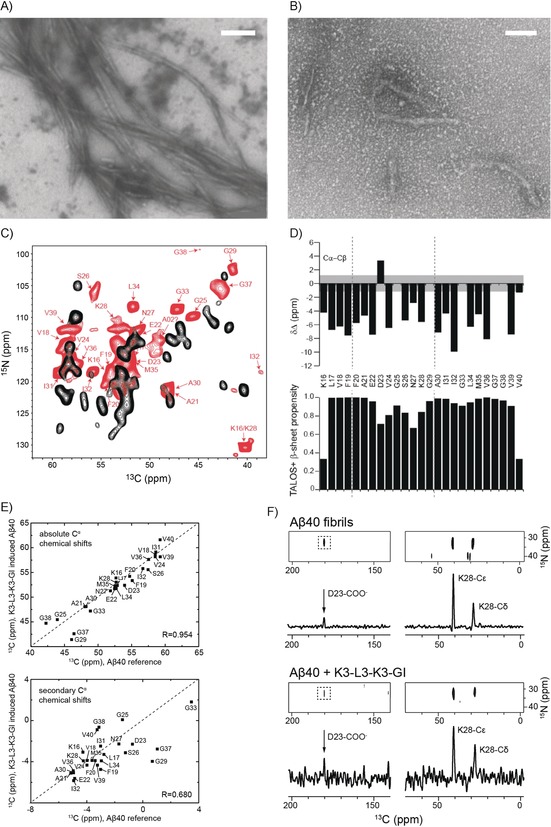
Structural characterization of K3‐L3‐K3‐GI‐induced Aβ40 aggregates. A, B) TEM images of Aβ40 in the absence (A) and presence (B) of an equimolar amount of K3‐L3‐K3‐GI. Scale bar: 200 nm. C) 2D ^13^C,^15^N MAS solid‐state NMR correlation spectra obtained for K3‐L3‐K3‐GI‐induced Aβ40 aggregates (red). To prepare the sample, a 20 μm solution of monomeric Aβ40 was incubated with a 1.23‐fold molar excess of K3‐L3‐K3‐GI. For reference, the correlation spectrum obtained for Aβ40 fibrils is shown in black. To prepare the Aβ40 fibril sample, monomeric Aβ40 was grown using 5 % seeds, following the protocol described by Lopez and co‐workers.^17^ (D) Cα–Cβ chemical shift differences for K3‐L3‐K3‐GI‐induced Aβ40 aggregates (top). TALOS+^[19]^‐predicted secondary structure propensity (bottom). E) Correlation of the NMR chemical shifts observed for K3‐L3‐K3‐GI‐induced Aβ aggregates and Aβ fibrils. On top and bottom, correlations for the absolute and secondary Cα chemical shifts are shown. Secondary Cα chemical shifts indicate differences from random‐coil chemical shifts. The correlation coefficient is on the order of *R*=0.954 and *R*=0.680, respectively. The correlation coefficient is very high, indicating that the conformation in the two different preparations is surprisingly similar. F) 2D ^13^C,^15^N TEDOR MAS solid‐state NMR spectra for Aβ40 fibrils (top) and K3‐L3‐K3‐GI‐induced Aβ40 aggregates (bottom). Only the spectral region containing amino side chain nitrogen chemical shifts is shown. For both samples, a long‐range correlation peak between Nϵ of Lys‐28 and the carboxylic carbon atom of Asp‐23 is observed, indicating that a salt bridge is formed in the K3‐L3‐K3‐GI‐induced Aβ40 aggregates. The relative intensity of the long‐range NH_3_–COO^−^ cross peak appears to be larger in the K3‐L3‐K3‐GI/Aβ40 sample, indicating that this structure is presumably more compact.

The K3‐L3‐K3‐GI‐induced Aβ40 aggregates were analyzed by TEM and solid‐state NMR spectroscopy. TEM indicates the presence of mainly amorphous aggregates while short Aβ40 protofibril‐like assemblies were also observed (Figure 6 A, B). At first sight, the ISM‐induced aggregates appear heterogeneous. However, solid‐state NMR experiments yielded high‐resolution spectra, indicating that the ISM–Aβ40 co‐assemblies are homogeneously structured (Figure 6 C). In fact, the spectral resolution obtained for these aggregates is very similar to the resolution achieved for Aβ40 fibrils that were obtained after several rounds of seeding.^17^ We performed chemical shift assignments to identify the residues of Aβ40 that are part of the core of the ISM‐induced aggregates (Table S6). Based on ^13^Cα and ^13^Cβ NMR chemical shifts, we predicted the β‐strand secondary structure elements for K3‐L3‐K3‐GI‐induced Aβ40 aggregates (Figure 6 D). We found that the same residues as in an Aβ amyloid fibril are immobilized and involved in the β‐sheet core.^17^ Furthermore, a comparison of the NMR secondary chemical shifts for the two preparations shows a high degree of similarity (Figure 6 E), indicating that the fold of the two aggregates is rather related. In addition, we observed a TEDOR cross‐peak involving the carboxyl group of residue Asp‐23 and the ϵ‐amino group of Lys‐28 (Figure 6 F), suggesting the formation of a salt bridge between the two residues. This interaction is a characteristic feature of all Aβ40 fibril structures determined thus far,^18^ and confirms that also K3‐L3‐K3‐GI‐induced Aβ40 aggregates adopt a β‐arch‐like fold upon interaction with a substrate amyloid in the solid state. Even though the morphology of the K3‐L3‐K3‐GI‐induced Aβ40 aggregates is rather different from that of Aβ40 amyloid fibrils, we conclude that both complexes adopt a similar β‐sheet/turn/β‐sheet molecular architecture. However, a more detailed structural analysis is necessary to characterize the exact structural features of ISM‐induced Aβ aggregates.

## Discussion

We have found that the ISM inhibitor R3‐GI can adopt a β‐like fold in solution. At the same time, we observed supramolecular, granule‐like structures by DIC and fluorescence microscopy, which suggests that R3‐GI may undergo LLPS. STD NMR and FRAP experiments implied that the peptide exchanges between a monomeric and a high‐molecular‐weight soluble aggregated state. MAS solid‐state NMR experiments showed that the β‐arch‐like architecture of an Aβ40 amyloid fibril with the characteristic salt bridge between Asp‐23 and Lys‐28 is preserved in the solid state in the amyloid–inhibitor complex. R3‐GI peptides thus form highly dynamic assemblies that provide a suitable surface for sequestration of Aβ40.

Conventional inhibitors, following the classical key–lock or induced‐fit principle, target specific structural motifs, for example, a deep hydrophobic binding pocket.^20^ To be potent, these inhibitors have to be very specific with a high binding affinity. When the binding specificity is reduced, ligands can exploit multivalent interactions to yield increased avidity. Bacterial toxin inhibitors, for example, are based on multivalent scaffolds.^21^ Similarly, most protein–carbohydrate interactions are multivalent to compensate for their low affinities. Multivalency has been employed to trigger signal transduction by inducing receptor clustering,^22^ and to design amyloid Aβ inhibitors^23^ where small‐molecule inhibitors have been covalently coupled to chaperones to increase their steric bulk. We suggest that R3‐GI and related ISMs exploit multivalency by self‐association. The correct spatial arrangement of side chains allows them to efficiently capture Aβ40 and direct it into off‐pathway non‐toxic aggregates.^24^

**Figure 7:**
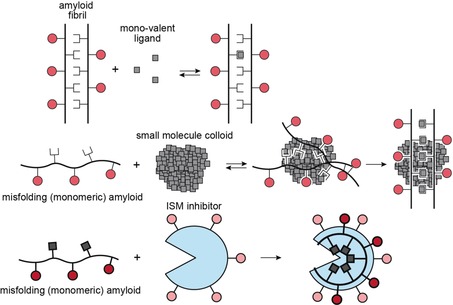
Different mechanistic pathways of ligand binding to amyloidogenic proteins. Top: mono‐valent ligands (e.g., small molecules) bind to a specific site in a particular peptide strand. Middle: in colloids, the local small‐molecule concentration is increased, facilitating ligand binding. Amyloid peptides are recruited into the colloid. Bottom: the IAPP‐derived ISM inhibitor R3‐GI is suggested to self‐assemble and recruit Aβ40 into non‐toxic aggregates. Circles and squares represent hydrophilic and hydrophobic side chains, respectively. The inner core of the ISM is shown in light blue to indicate its hydrophobic environment.

Figure 7 schematically illustrates different amyloid self‐assembly inhibition mechanisms involving classical single‐site binding, together with intervention strategies that employ colloid formation or the here suggested ISM‐induced LLPS‐like process. Single‐site binding events are the classical paradigm for a drug–enzyme complex. Because of the low affinity and the lack of deep binding pockets, this class of inhibitors is not very effective for amyloids (Figure 7, top). Nevertheless, it has been shown that multi‐ligand interactions of tramisprosate with monomeric Aβ42 can prevent amyloid oligomer formation.^25^ Hydrophobic small molecules can form colloids that resemble protein liquid droplets in size.^7^ These small‐molecule colloids are, however, unspecific inhibitors, as they interact promiscuously with hydrophobic regions of a protein and eventually induce protein unfolding. We have shown previously that NSAIDs (non‐steroidal anti‐inflammatory drugs) such as sulindac sulfide can bind into hydrophobic cavities of amyloid fibrils, and stabilize aggregates.^8^, ^26^ The NSAID accelerates Aβ peptide aggregation by recruiting Aβ peptides into its colloidal core (Figure 7, center).

Our data suggest that ISMs may act via a similar mechanism: ISMs are active at very low concentrations and make use of multivalent interactions, which may dramatically increase avidity (Figure 7, bottom). Multivalent interactions between an inhibitor molecule and an amyloid fibril have been exploited by Wall and co‐workers,^27^ who designed an α‐helical peptide with a lysine basic side chain to interact with negatively charged residues exposed on the surface of amyloid structures. These inhibitors are used as imaging reagents and stabilize a fibrillar fold. In contrast, ISMs prevent amyloid formation and redirect Aβ40 into an off‐pathway aggregate that lacks cellular toxicity. Importantly, R3‐GI self‐assembly is likely required but not sufficient for its amyloid inhibitor function. In fact, macroscopic particles are observed in solution for both the non‐inhibitor G3‐GI and the inhibitor R3‐GI in DIC experiments. However, only R3‐GI yields long‐range contacts in NOESY experiments, supporting the idea that the specific loop‐containing structure of R3‐GI identified here is essential for its potent inhibitory function.1b Notably, Aβ40 in solution exhibits a conformational preference for β‐arch‐like structures as well.^28^ Thus, R3‐GI and related ISMs may exert their inhibitory function by providing a structural template to which specific, amyloidogenic Aβ40 conformers can adhere.

## Conclusion

The highly potent inhibitor function of the ISMs renders them well suited templates for the development of anti‐amyloid drugs.1b, 24b Our findings provide a molecular basis for understanding their function and should thus assist in the design of novel potent anti‐amyloid drugs. In addition, they may contribute to elucidating the mechanism of previously reported self‐assembling peptide inhibitors designed to mimic surfaces involved in self‐ or cross‐amyloid interactions, for which the mode of action is thus far not understood.^29^

## Conflict of interest

All authors except for A.K., who is a coinventor in a patent application on ISMs reported in this work, declare no conflict of interest.“

## Supporting information

As a service to our authors and readers, this journal provides supporting information supplied by the authors. Such materials are peer reviewed and may be re‐organized for online delivery, but are not copy‐edited or typeset. Technical support issues arising from supporting information (other than missing files) should be addressed to the authors.

Supplementary
