# Supplementary material for: Structural Insight into IAPP‐Derived Amyloid Inhibitors and Their Mechanism of Action
Source: Angew Chem Int Ed Engl. 2020 Jan 28;59(14):5771–81. doi: 10.1002/anie.201914559 (PMC7154662; doi:10.1002/anie.201914559)
Supplement: Supplementary file 1 — Supplementary [file ANIE-59-5771-s001.pdf]

Angewandte Chemie
Eine Zeitschrift der Gesellschaft Deutscher Chemiker

Supporting Information

Structural Insight into IAPP-Derived Amyloid Inhibitors and Their Mechanism of Action

Zheng Niu, Elke Prade, Eleni Malideli, Kathleen Hille, Alexander Jussupow, Yonatan G. Mideksa, Li-Mei Yan, Chen Qian, Markus Fleisch, Ana C. Messias, Riddhiman Sarkar, Michael Sattler, Don C. Lamb, Matthias J. Feige, Carlo Camilloni, Aphrodite Kapurniotu, and Bernd Reif**

anie_201914559_sm_miscellaneous_information.pdf

Materials and Methods

Peptide synthesis and sample preparation for NMR

Peptides were synthesized by solid phase synthesis and provided as films evaporated from hexafluoroisopropanol (HFIP) in the Eppendorf tube as described in Andreetto et al. [1]. The R3-GI peptide was dissolved in 10 mM sodium phosphate buffer, pH 7.4 (containing 1% v/v HFIP and 10% v/v D_2O), to a final concentration of 1.5 mM. The other peptide samples were prepared similarly. The final concentration of R3-G, R3-I and R3 was 1 mM, respectively. Experiments involving G3-GI were carried out at a concentration of 0.5 mM.

Sample preparation for DLS, TEM and DIC

The R3-GI peptide was dissolved in 10 mM sodium phosphate buffer, pH 7.4 (containing 1% v/v HFIP) to a final concentration of 50 μM for DLS measurement. The TEM image has been captured immediately after dissolution of 500 μM R3-GI peptide into 10 mM sodium phosphate buffer, pH 7.4 (containing 1% v/v HFIP) without filtration. The DIC micrographs of 500 μM Fluos-R3-GI, 500 μM Fluos-K3-L3-K3-GI and 500 μM Fluos-G3-GI have been imaged immediately after dissolution into 10 mM sodium phosphate buffer, pH 7.4 (containing 1% v/v HFIP) without filtration. The Fluor-647-Aβ40 was ordered from AnaSpec, and the peptide is labeled on the N-terminus with HiLyte™ Fluor 647. For the substrate interactions, Fluor-647-Aβ40 and Fluos-labeled ISMs were incubated at room temperature for 20 min before capturing images. The GFP filter cube (Exc./BP 470/40; Em./BP 525/50) and the Y5 filter cube (Exc. BP 620/660; Em. BP 700/75; DC 660) were used to capture the fluorescence images.

FRAP and FCS experiments

FRAP and FCS measurements were performed on a home-built confocal laser scanning microscope, as described elsewhere [2]. An oil immersion objective (Apo-TIRF 100x Oil/NA 1.49, Nikon) was used for all measurements. A 470 nm pulsed diode laser (LDH-P-C-470, Picoquant) was used for excitation. Fluorescence emission was detected using an avalanche photodiode (COUNT-100B, Laser Components) after a 520/40 emission filter. All measurements were carried out at 22 °C. For FCS, a mixture of 454 μM R3-GI and 454 nM Fluos-R3-GI was added to the coverslip (Labtek II chambered coverglass, Thermo Scientific) and washed once with 10 mM sodium phosphate buffer, pH 7.4 (containing 1% v/v HFIP). The focal spot was positioned either in solution or inside a droplet. To avoid spherical aberrations in the focal spot, care was taken to always position it near the center of the 60 x 60 μm scan range. Fluorescence emission was measured for 5 min using 2 μW laser power (measured before the back aperture of the objective).

For FRAP, a 500 μM Fluos-R3-GI sample was added to the coverslip in the same way as for FCS. The 470 nm laser power was further attenuated to 0.2 μW. 50 frames were recorded before bleaching (100 x 100 pixels in a 2.0 x 2.0 μm area, 100 ms frame time). A 405 nm pulsed diode laser (LDH-P-C-400B, Picoquant) was then used to bleach the sample at 50 μW for 2000 frames with the same scan parameters. Imaging with the 470 nm laser was immediately resumed after the 405 nm laser was turned off, and continued until the fluorescence intensity reached a plateau.

Raw photon data was processed and analyzed with the software package PIE analysis with MATLAB (PAM) [3]. The auto-correlation functions (ACFs) from FCS measurements were fitted with a multi-component free diffusion model:

$$G(\tau) = \frac{\gamma_{FCS}}{\langle N \rangle}\left(1 + \frac{T}{1-T}\exp(-\tau/\tau_T)\right)\sum_{i=1}^{M}\frac{1}{(1+4D_i\tau/\omega_r^2)(1+4D_i\tau/\omega_z^2)^{\frac{1}{2}}}$$

where τ is the time shift, $\gamma_{FCS} = 2^{-\frac{3}{2}}$ the shape factor for a 3D Gaussian, $\langle N \rangle$ is the average number of molecules in the focal volume, $T$and τ_T are the triplet fraction and time, M is the number of diffusion components (restricted to 1 or 2 depending on the sample), and D_i is the diffusion coefficient of each component. ω_r and ω_z are the lateral and axial 1/e^2-radii of the focal volume and were determined to be 144 nm and 602 nm respectively by fitting the ACF of an ATTO488 calibration sample with a fixed diffusion coefficient of 373 μm^{2}s^{-1} [4].

The FRAP analysis of intensity images exported from PAM was done using the FRAP calculator macro in ImageJ [5].

NMR Spectroscopy

All NOESY NMR experiments were performed using a Bruker 900 MHz Avance III spectrometer, equipped with a triple resonance cryo-probe, setting the temperature to 4 °C. In all experiments, a NOESY mixing time of 300 ms was employed. The experiments were processed with TopSpin and analyzed with CcpNmr [6]. The peptide was dissolved in 10 mM sodium phosphate buffer, pH 7.4 (containing 1% v/v HFIP and 10% v/v D_2O), to a final concentration described above.

The DOSY experiments were acquired on a Bruker Avance III 500 MHz spectrometer equipped with a cryogenic probe. A series of diffusion spectra were recorded with gradient (along z axis) strength varying from 1.07 to 48.15 G/cm at 277 K, 283 K and 293 K, respectively. The sine shaped gradient pulses each had a duration of 1600 μs. The diffusion interval was set to 0.23 s. The DOSY data were fit using the equation [7]

$$ln\frac{I}{I_0} = -\gamma^2\delta^2G^2D\left[\Delta + \left(\frac{4\delta}{3} + \frac{3\tau}{2}\right)\right]$$

where I and I_0 correspond to the intensity of the NMR signal at a given gradient strength and at the minimum gradient strength of 2 %, respectively. γ refers to the gyromagnetic ratio, δ is the length of the gradient pulse which is set to 1.6 ms. The gradient strength was varied between 1.07 G/cm and 48.15 G/cm. D corresponds to the diffusion constant. In the experiment, the diffusion delay Δ was set to 0.23 s. The gradient recovery delay τ was adjusted to 400 µs. Here we used the mixture sample containing 0.5 mM labeled and 0.5 mM non-labeled R3-GI peptide, which was dissolved in 10 mM sodium phosphate buffer, pH 7.4 (containing 1% v/v HFIP and 10% v/v D_2O).

STD NMR experiments of 1.25 mM R3-GI in 10 mM sodium phosphate, pH 7.4 containing 1% (v/v) HFIP and 10% (v/v) D_2O were carried out on a Bruker Avance III 600 MHz spectrometer equipped with a cryogenic probe at 283 K, 298 K and 308 K, respectively. 1D proton experiments were performed using a WATERGATE pulse sequence with 32 k time domain points and 128 scans. STD spectra were recorded using an interleaved pulse program with on- and off-resonance irradiation. Saturation was achieved by a chain of Gaussian-shaped pulses of 49 ms each, with on-resonance saturation at -1 ppm and off-resonance at -100 ppm with 2 seconds total saturation time, using 2048 scans and 32 k time domain points.

To prepare the Aβ40 fibril sample, monomeric Aβ40 was grown using 5 % seeds, following the protocol described in Lopez et al. [8]. Solid-state NMR spectra were recorded at a MAS rotation frequency of 15.5 kHz MAS, setting the effective sample temperature to 10 °C. Experiments were recorded at an external field strength of 17.6 T, corresponding to a ^{1}H Larmor frequency of 750 MHz.

Ensemble modelling

R3-GI ensembles were generated using Metadynamic Metainference [9] using NOE derived distances as the experimental information. Simulations were performed with GROMACS [10], using PLUMED2 [11] and the PLUMED-ISDB [12] module that implements Metadynamic Metainference and multiple experimental restraints. In Metadynamic Metainfence multiple replicas of a system in the same experimental conditions are run in parallel. The replicas are coupled by a potential that is applied on the replica-averaged back calculated NOE-distances and restrains the calculated observables to their experimental values within an error that is estimated on-the-fly by Gibbs sampling. Furthermore, the sampling of the replicas is enhanced by multiple-walkers parallel-bias metadynamics, where multiple collective variables are biased at the same time and the history-dependent bias is shared among the replicas.

Metadynamic Metainference simulations were performed using 12 replicas. Briefly, the peptide was prepared with Pymol in an extended conformation, including the G17 and I19 N-methylation and the C-terminus amine group. Two starting structures were generated setting the peptide bond dihedral for G17 to trans and cis, respectively. The peptides were modelled using the Amber99SB force-field in TIP4P-D water [13]. Van der Waals and Coulomb interactions were implemented with a cut-off at 0.9 nm, and long-range electrostatic effects were treated with the particle mesh Ewald method on a grid with a mesh of 0.12 nm. All simulations were carried out in the isothermal and isobaric ensemble by thermosetting the system using a stochastic velocity rescaling and controlling the pressure using the Parrinello-Rahman barostat. The structures were solvated with 2500 water molecules and ions were added to neutralize the total charge. Two preliminary 10 ns long simulations were run for each structure to equilibrate the system and obtain independent starting configurations.

Parallel bias metadynamics was applied to five collective variables (CVs) namely, the helix content, the anti-parallel beta content, the radius of gyration carbons and two AlphaBeta collective variables defined as one half of the sum over all residues of one plus the cosine of either the ψ or the χ_1 angles for all residues, respectively. Gaussians deposition was performed with an initial rate of 2.5 kJ/mol/ps, the sigma of the Gaussian was adaptively estimated evaluating the fluctuations of the CVs on a time window of 5 ps. The height of the Gaussians was rescaled as in well-tempered metadynamics with a bias-factor of 20. Each replica was run for 500 ns.

The Metainference force constants for the NOE derived distances (only inter-residues distances were employed consisting in 221 and 35 restraints for the first and the second conformers, respectively), were determined using a Gaussian noise model for each experimental value. In this case the Metainference energy is:

$$E_{MI} = E_{FF} + \frac{k_B T}{2} \sum_{r,i} \frac{\left[d_i - f_i(\boldsymbol{X})\right]^2}{\left(\sigma_{r,i}^B\right)^2 + \left(\sigma_{r,i}^{SEM}\right)^2} + E_\sigma$$

where the force field of standard MD simulations $E_{FF} = -k_B T \sum_{r=1}^{N} \log p(X_r)$ is modified by *i)* a series of (harmonic) *data-restraints*, which enforce the agreement of the replicas with the ensemble-averaged data (d_i are the experimentally derived distances and $f_i(\boldsymbol{X})$ those calculated as average over the replicas), and *ii)* a series of error restraints, $E_\sigma = k_B T \sum_{r,i} \left\{-\log p\left(\sigma_{r,i}^B\right) + 0.5 \log\left[\left(\sigma_{r,i}^B\right)^2 + \left(\sigma_{r,i}^{SEM}\right)^2\right]\right\}$. Here $\sigma_{r,i}^{SEM}$ was automatically estimated as the maximum value of the standard-error of the mean in a time window of 200 steps, while $\sigma_{r,i}^B$ was estimated on-the-fly using a Gibbs sampling scheme.

The sampling of the 12 replicas was combined resulting in the ensemble of conformations were the statistical weight of each conformation is obtained by a simple reweighting scheme based on the final metadynamics bias B. The weight of a conformation ***X*** is given by $w=exp(+B(\mathbf{X})/k_BT)$, consistently with the quasi static behavior at convergence of well-tempered metadynamics.

Coarse-grained modelling of R3-GI oligomers

To investigate the interaction of the peptides with each other, over 100 µs of coarse grain simulations were performed using GROMACS. An initial peptide model was created based on a representative structure from the atomistic ensemble. The Martini 2.2 force field was used to describe the peptide interaction together with the standard Martini water model [14]. Additionally, an elastic network was applied to conserve structural information.

An initial 76.1 µs run was performed containing 54 peptides in a cubic box with an initial size length of 22.67 nm as an isothermal-isobaric (NpT) ensemble using 20 fs time steps, a temperature of 300 K and a pressure of 1 bar. The v-rescale thermostat was used with a coupling constant τ_t = 1.0 to control the temperature. The pressure was semi-isotropic coupled with a coupling constant of τ_p = 20.0 ps and a compressibility of $\chi = 3.0 \times 10^{-4}$ bar^{-1} using the Parrinello-Rahman barostat. Due to the high amount of positive charge in R3-GI, PME was used for the calculation of the electrostatic interactions. To test whether our model was sensitive enough to discriminate between different sequences and structures, we performed two 16.5 and 18.7 µs long simulations with 54 $(SG)_{10}S$ peptides as well as two 10 µs long simulations with 54 G3-GI peptides with and without the elastic network containing the structural information of R3-GI.

Far-UV CD measurements

Measurements were carried out using a Jasco 715 spectropolarimeter. Spectra were collected between 195 and 250 nm at 0.1 nm intervals with a response time of 1 second at room temperature and each spectrum is the average of 3 spectra. CD studies were performed in freshly made peptide solutions at the indicated concentrations in aqueous 10 mM sodium phosphate buffer, pH 7.4, containing 1% HFIP as previously described [15]. Peptide stock solutions in HFIP used for all CD measurements were freshly made. The buffer was always subtracted from the CD spectra of the peptide solutions.

Fluorescence spectroscopic titrations

A JASCO FP-6500 fluorescence spectrophotometer was used and titrations were performed as previously described [15]. Briefly, excitation was at 492 nm and fluorescence emission spectra were recorded between 500 and 600 nm. A freshly made solution of Fluos-R3-GI (5 nM) was titrated with R3-GI in 10 mM sodium phosphate buffer, pH 7.4 (1% HFIP) at room

temperature within 2-5 min following solution preparation. The determined app. K_d is means (±SD) from three binding curves [15].

Cross-linking and NuPAGE

Cross-linking studies were performed as previously described [15]. Briefly, R3-GI was dissolved at 500 µM in aqueous 10 mM sodium phosphate buffer, pH 7.4, and incubated for the indicated time points at room temperature, cross-linked with aqueous glutaraldehyde (25%) (Sigma-Aldrich), precipitated with aqueous trichloroacetic acid (TCA) (10%) and pellets were dissolved in NuPAGE sample buffer and subjected to NuPAGE electrophoresis in 4-12% Bis-Tris gels with MES running buffer. Equal amounts of peptide were loaded in all lanes. Coomassie blue staining was performed following standard protocols.

Dynamic light scattering experiments

DLS measurements were performed at 658 nm using a DynaPro NanoStar (Wyatt Technology) equipped with a 90° scattering angle detector. Before the measurement, samples were centrifuged at 12000 rpm for 2 min using a table centrifuge (Heraeus Fresco 17 Centrifuge, Thermo Scientific) to remove aggregates. The sample volume was 60 µL, employing single use cuvettes (UVette 220-1600 nm, Eppendorf). Experiments were performed at 25 °C and averaged over 10 measurements, setting the acquisition to 15 s with auto-attenuation turned on.

Thioflavin T (ThT) binding assay

Effects of peptides on amyloidogenesis of Aβ40 were determined using a previously established ThT binding assay as described [15]. Briefly, Aβ40 alone or in mixture with ISM (Aβ40/ISM ratio 1/1) was incubated in ThT assay buffer consisting of aqueous 50 mM sodium phosphate buffer, pH 7.4, containing 100 mM NaCl and 1% HFIP at room temperature as described. At the indicated time points, aliquots were gently mixed with a solution consisting of 20 µM ThT in 0.05 M glycine/NaOH, pH 8.5. ThT binding was determined immediately by measuring fluorescence emission at 486 nm following excitation at 450 nm using a 2030 Multilabel Reader VictorX3 (PerkinElmer Life Sciences).

Transmission electron microscopy (TEM)

To prepare samples for TEM, 10 µL of each sample was placed on the EM grid for 1 min, followed by a drying procedure with filter paper. The grid was subsequently washed three times by adding a drop of water for 3 s, and drying it each time with filter paper. For staining, 10 µL of a 1% uranyl acetate solution was added for 30 s. The excess of the solution was dried with filter paper. Grids were purchased from Electron Microscopy Sciences (Hatfield,

PA 19440, USA; Formvar/Carbon 300 mesh copper coated). Samples were measured immediately employing an EM 10 CR (Zeiss, Germany).

MTT reduction assay

The cell damaging effects of Aβ40 aggregates and its mixtures with the ISMs were determined in combination with the ThT binding assays using cultured PC12 cells in 96-well plates as recently described [15]. Briefly, 7 days aged solutions of Aβ40 alone or its mixtures with ISMs (solutions from the ThT binding assays) were added to the cells at the indicated final concentrations (after being diluted with cell medium) and incubated with the cells for ~20 h (37°C, humidified atmosphere with 5% CO_2). Cell damage was assessed by determination of the cellular reduction of the MTT dye as described [15].

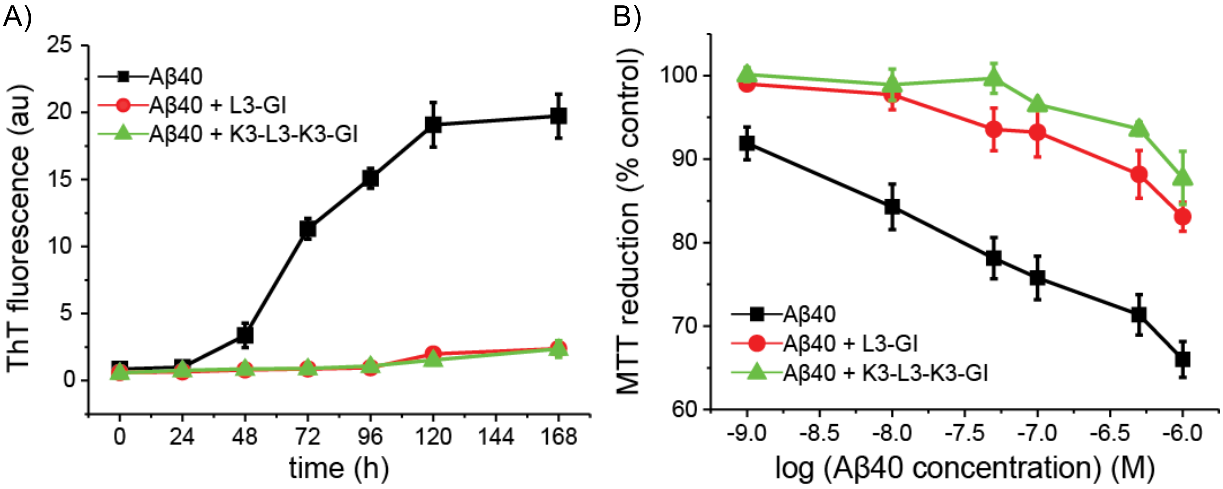

Supporting Figure 1. Effects of K3-L3-K3-GI on Aβ40 amyloid self-assembly and cell toxicity. *(A) Amyloid self-assembly of Aβ40 alone and its mixtures (1:1) with K3-L3-K3-GI or L3-GI (control) determined using the ThT binding assay (means (±SEM), 3 assays). (B) Effects on Aβ40 cytotoxicity. 7 days aged solutions from (A) were added to PC12 cells; cell damage was assessed by the MTT reduction assay (means (±SEM), 3 assays (n=3 each)).*

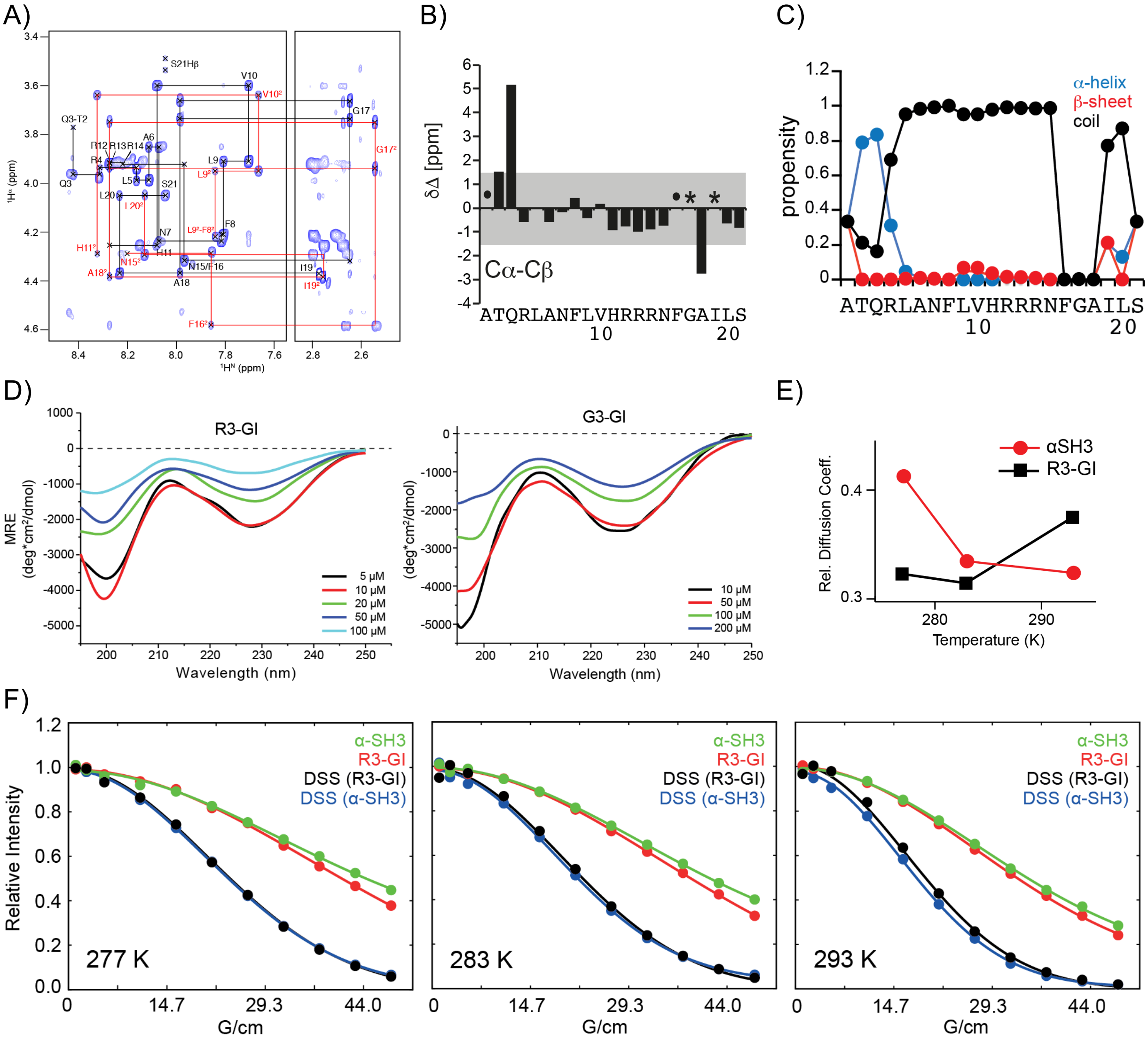

Supporting Figure 2. Conformational analysis of R3-GI and G3-GI in solution. *(A) 2D ^{1}H-^{1}H NOESY of a 1.5 mM solution of R3-GI, including assignment of residues T02-S21. A second conformer is observed for residues F8-H11 and residues N15-L20. Sequential assignments are represented for the HN-Hα spectral region for conformer 1 (black) and conformer 2 (red, superscript "2"). Experiments were recorded at 4 °C and 21.1 T (900 MHz), employing a NOESY mixing time of 300 ms. (B) ^{13}C difference chemical shifts (ΔδCα-ΔδCβ). A negative value indicates β-sheet structure, whereas a positive value is found for residues involved in α-helical structure. The grey bar (±1.4 ppm) indicates random structure according to Wishart et al. [16]. Values within this range are not considered a significant amount of secondary structure. ^{13}C spectra were obtained at carbon natural abundance. • incomplete chemical shift assignment for this residue. * no random coil chemical shift reference values for N-methylated amino acids available. (C) Secondary structure propensities according to TALOS+ [17]. Population of random coil, β-sheet and α-helices is indicated by black, red and blue lines, respectively. (D) CD spectra of R3-GI (left) and G3-GI (right) recorded at the indicated concentrations (pH 7.4). The observed concentration dependence is consistent with peptide oligomerization. (E) Temperature dependent diffusion coefficients from DOSY experiments recorded for R3-GI. DOSY experiments for α-spectrin SH3 domain and* 4,4-dimethyl-4-silapentane-1-sulfonic acid *(DSS) have been measured for reference. (F) DOSY diffusion experiments of R3-GI recorded at a temperature of 4 °C, 10 °C and 20 °C. For comparison, the 7.229 kDa globular protein domain α-spectrin SH3 (R_H = 15.3 Å) has been measured under the same conditions. In all samples,* 4,4-dimethyl-4-silapentane-1-sulfonic acid (DSS) *has been added as an internal standard to account for the viscosity of the solution. Comparison of the diffusion data of R3-GI with the diffusion coefficient of the 7.23 kDa α-spectrin SH3 domain yields an apparent diffusion coefficient for R3-GI that is ca. 1.3-fold smaller (at a temperature of 4 °C), suggesting that R3-GI exchanges between a monomeric and an aggregated state.*

A)

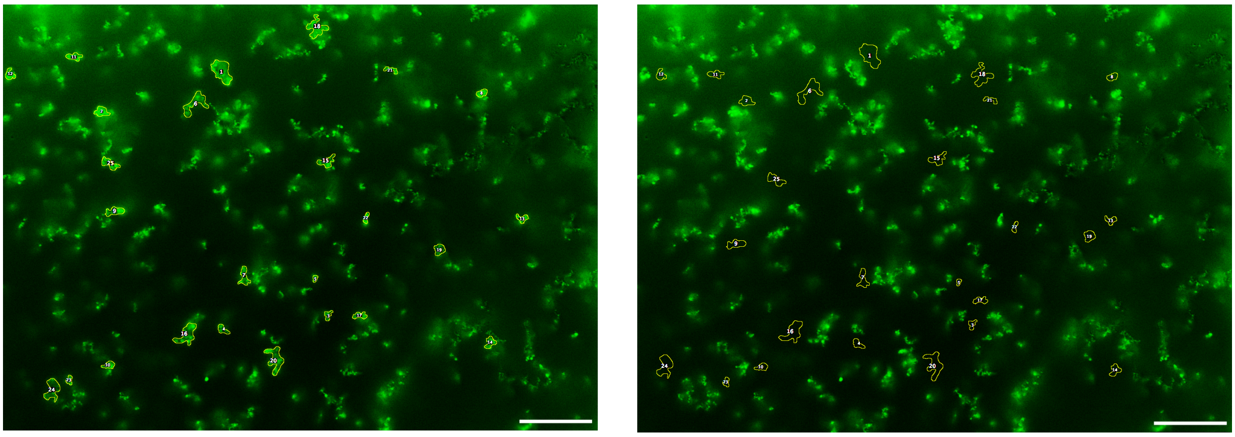

Structure	Area	Mean		Background	Area	Mean
1	0.331	12.871		1	0.331	6.069
2	0.107	18.174		2	0.107	6.382
3	0.052	9.555		3	0.052	2.592
4	0.068	9.245		4	0.068	2.6
5	0.026	10.745		5	0.026	3.453
6	0.243	10.244		6	0.243	5.538
7	0.116	11.447		7	0.116	3.553
8	0.065	16.453		8	0.065	6.346
9	0.121	13.213		9	0.121	3.967
10	0.068	11.561		10	0.068	3.414
11	0.088	15.161		11	0.088	6.337
12	0.068	17.433		12	0.068	7.796
13	0.065	12.678		13	0.065	3.818
14	0.087	11.027		14	0.087	3.773
15	0.12	11.463		15	0.12	3.913
16	0.25	10.637		16	0.25	3.012
17	0.073	11.765		17	0.073	3.113
18	0.232	14.222		18	0.232	4.993
19	0.098	8.059		19	0.098	3.75
20	0.258	10.513		20	0.258	2.722
21	0.047	12.755		21	0.047	4.588
22	0.041	14.773		22	0.041	3.55
23	0.044	10.279		23	0.044	3.774
24	0.211	7.974		24	0.211	3.374
25	0.118	13.663		25	0.118	4.248
Average intensity for peptide		12.2364		Average intensity for background		4.267

B)

sample	concentration	number of scans	Thr methyl S/N	normalized S/N
R3-GI	1.25 mM	128	3555,24	0,79
reference	60 μM	128	216,82	1,00

Supporting Figure 3. Estimation of the partitioning coefficient of the R3-GI solution. (A) *The top of the figure shows fluorescence microscopic images of R3-GI, with picked structures (left) and background (right) highlighted with orange lines. The scale bar in the figure denotes a length of 25 μm. The table below indicates the size of the area and the fluorescence intensity for each area element. The ratio in fluorescence intensities suggests a 2.9x enrichment of R3-GI in the aggregate structure in comparison to the solution. (B) NMR intensity analysis of the R3-GI peptide with respect to a reference peptide sample which is not prone to aggregation. R3-GI as well as the reference peptide (sequence GVAEPEQDCAVTSGE) contain a spectrally isolated threonine methyl resonance that is employed to*

quantify the signal-to-noise ratio. For the R3-GI peptide, the signal to noise ratio of the isolated Thr-CH3 peak amounts to 3555.24. For the reference peptide, the signal to noise of the single Thr-CH3 peak corresponds to 216.82. The ratio of concentrations amounts to a factor of 20.83. Otherwise, 1D-^{1}H spectra were recorded under identical conditions. The experimental intensity for the R3-GI peptide is thus ~21% smaller as expected. These results support the quantitative analysis of the fluorescence images in which we determined the partitioning coefficient of the R3-GI solution (Fig. S3).

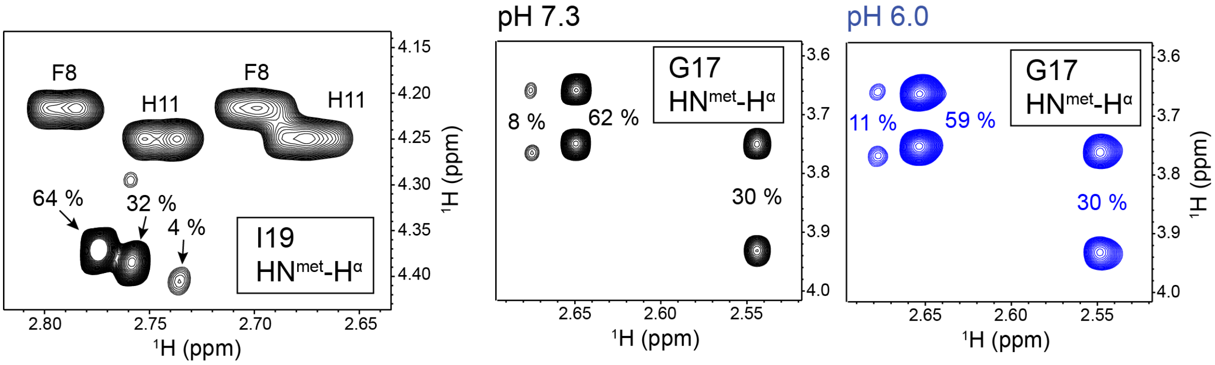

Supporting Figure 4. Cis-trans isomers induced by N-methylation at residue G17 and I19 of R3-GI*. The trans-trans, cis-trans and trans-cis conformers are on the order of 64 %, 32 % and 4 %, respectively. The cis-cis conformer is not high enough populated to be observed by NMR. We find that pH has a negligible effect on the population of the different cis-trans conformers.*

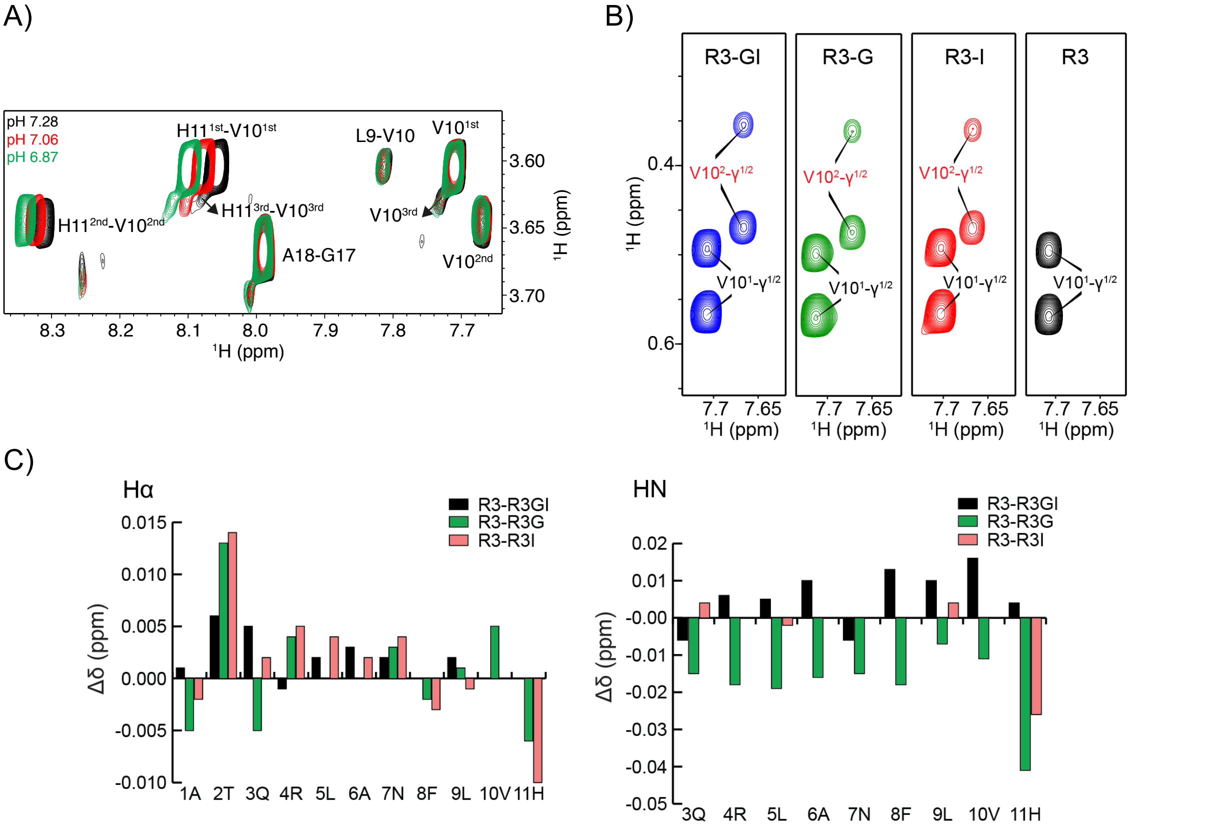

Supporting Figure 5. N-methylation induces the population of structured conformers at the N-terminal part of R3-GI*. A) Three sets of resonances are observed for residues F8-H11. Individual sets of resonances are only expected for conformers which convert on a timescale of seconds or longer. pH influences the H11-H^{N} resonance frequency, but does not impact on the relative population of the different conformers at the N-terminus. B) Downfield shifted resonance of V10-$\gamma^{1/2}$ suggests an interaction with an aromatic ring. In a subpopulation of the MD ensemble, the imidazole ring of H11 is in close proximity to the side chain of V10. C) H^{N}, Hα chemical shift differences for R3-GI, R3-G, R3-I with respect to R3.*

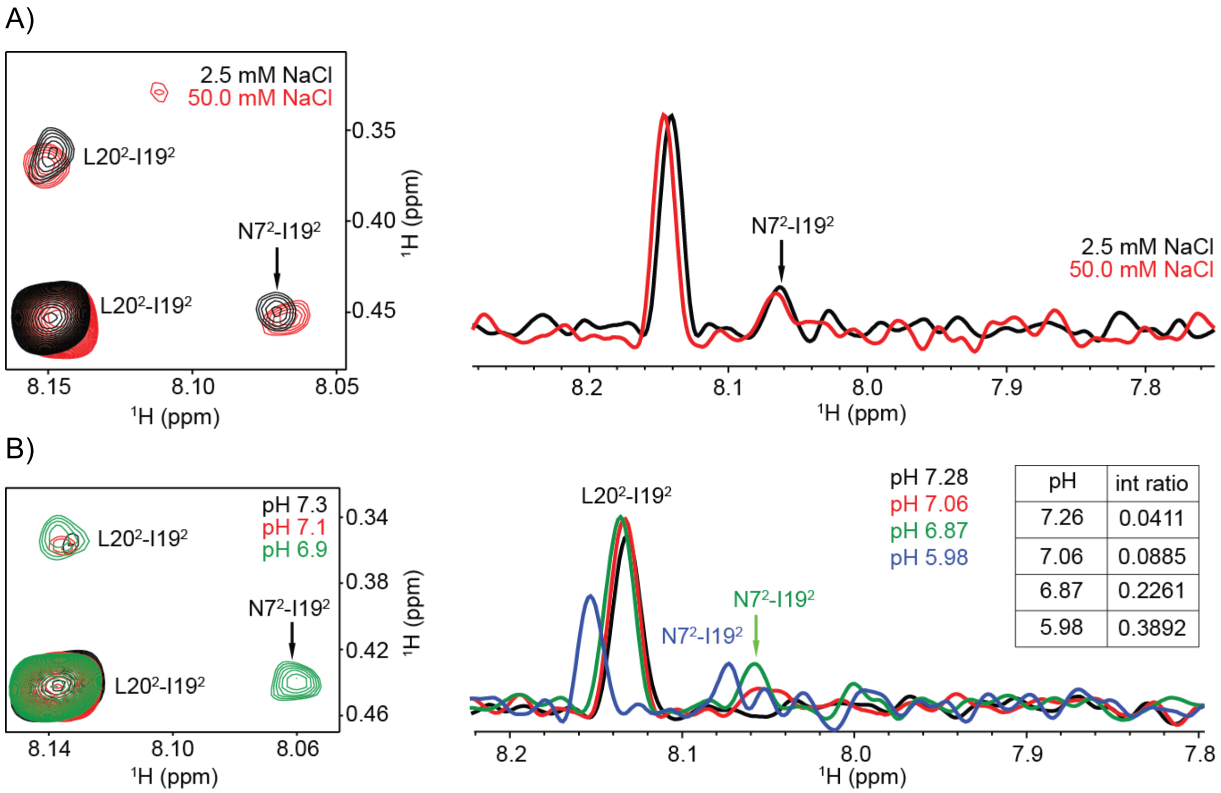

pH	int ratio
7.26	0.0411
7.06	0.0885
6.87	0.2261
5.98	0.3892

Supporting Figure 6. Influence of the salt concentration and pH on the exchange properties between soluble and aggregated forms of R3-GI*. Exchange is probed by inspection of the NOESY cross peak intensities. It is assumed that aggregated R3-GI has a significantly increased tumbling correlation time which in turn would result in higher NOESY cross peak intensities. A) ^{1}H-^{1}H NOESY spectrum recorded for a 500 μM solution of R3-GI, using a salt concentration of 2.5 mM (black) and 50 mM NaCl (red), respectively. In the two experiments the pH was kept at a value of 6.9. Change of the salt concentration does not impact NOESY cross peak intensities. B) ^{1}H-^{1}H NOESY spectrum recorded for a 500 μM solution of R3-GI, setting the pH to 5.98 (blue), 6.87 (green), 7.06 (red) and 7.23 (black). In all three experiments, the NaCl concentration was set to 2.5 mM. The inset shows the ratio of the cross peak intensities of the long-range contact $(N7\text{-}I19)^2$ versus $(L20\text{-}I19)^2$. At low pH, the relative ratio of the long-range cross peak intensity with respect to the sequential cross peak intensity is increased, suggesting that a larger fraction of R3-GI adopts a turn-like structure.*

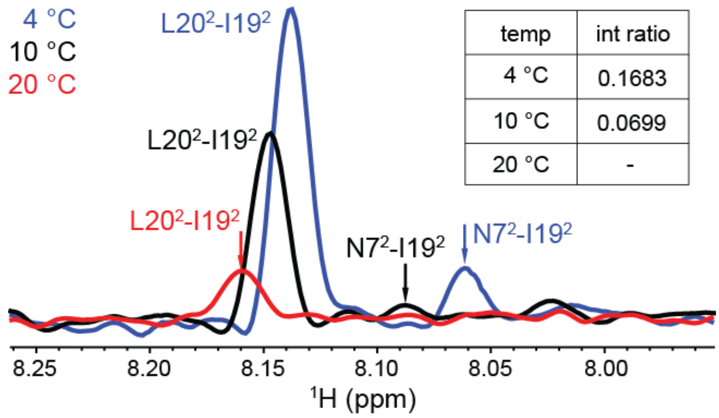

Supporting Figure 7. Temperature dependence of the long-range NOE (N7-I19)^2 cross peak in R3-GI*. Due to HN exchange, NOESY cross peak intensities decrease in general. However, the relative intensities of the long-range cross peak with respect to the sequential cross peak decreases at higher temperature, suggesting that the turn-like structure is more stable at low temperatures. The ^{1}H-1Ds represent the same cross section taken from the ^{1}H-^{1}H NOESY experiments as in the figures above.*

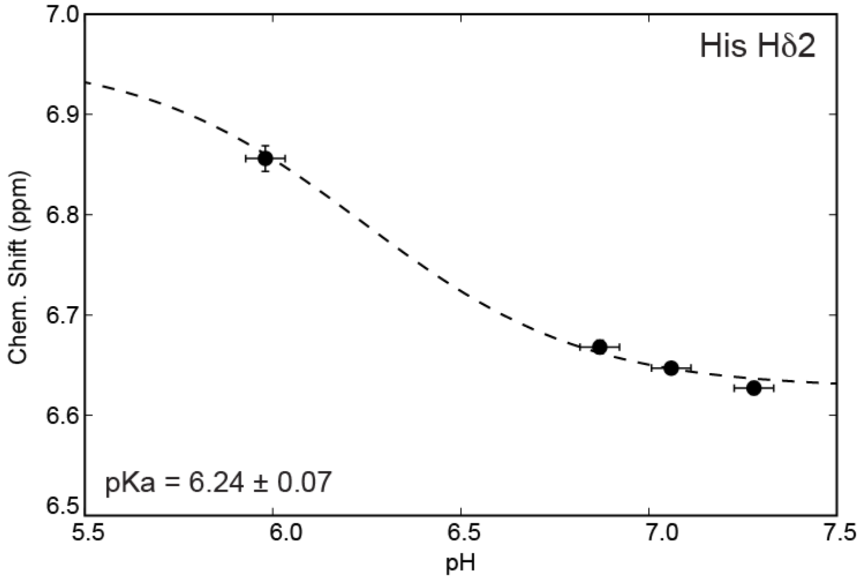

Supporting Figure 8. pH dependent chemical shift of His-11 Hδ2 of R3-GI*. The pKa of the histidine imidazole ring is on the order 6.23.*

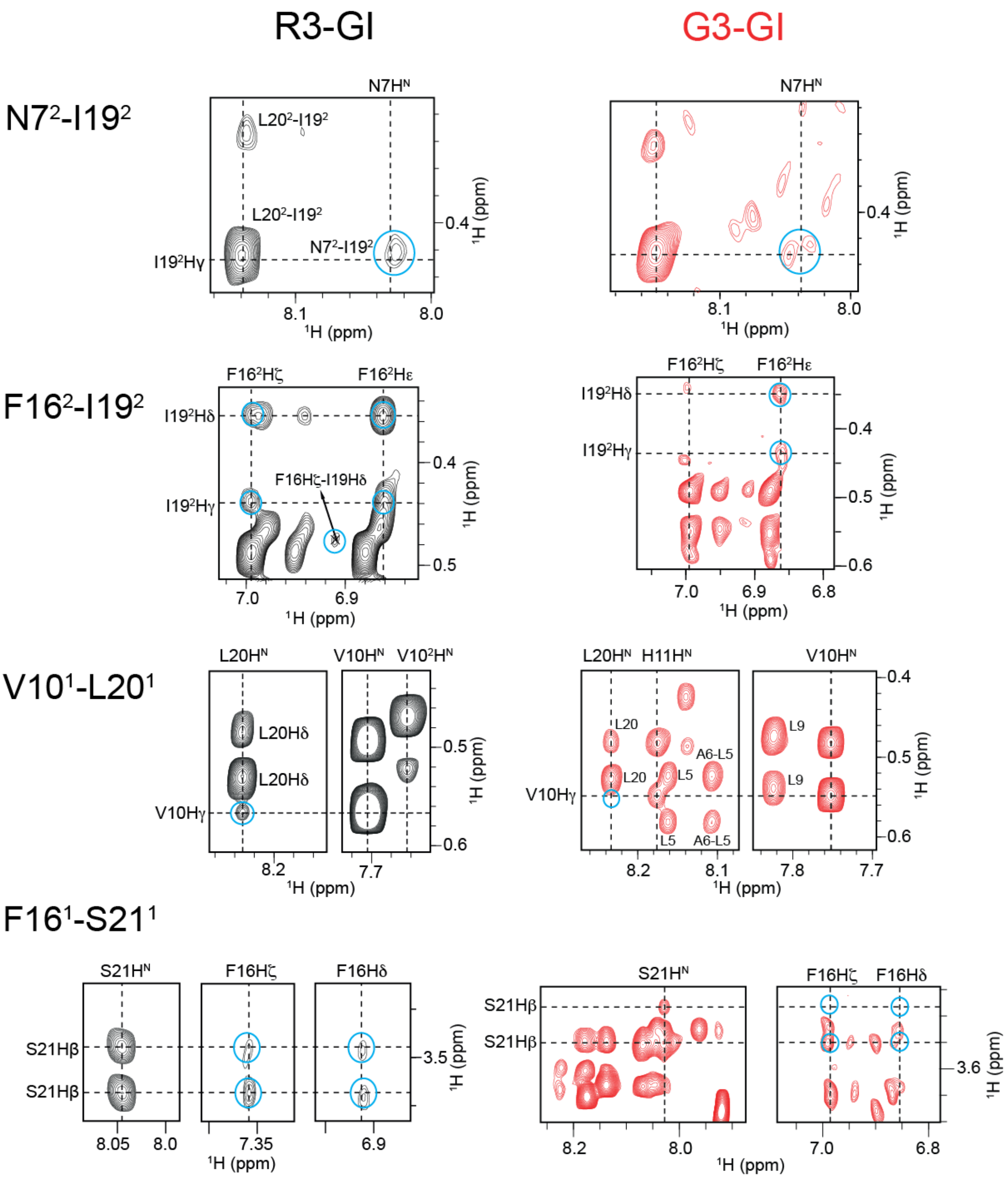

Supporting Figure 9. *Long range NOE contacts for the non-inhibitor G3-GI in comparison to R3-GI. Characteristic NOEs (or missing interactions in case of G3-GI) are indicated with blue circles. In general, long range NOEs are stronger for R3-GI. For conformer 1, e.g. the contact V10Hγ-L20H^N is missing. For conformer 2, e.g. the cross peak N7H^N-I19Hγ is very weak. This suggests that the loop is more stably formed for the ISM inhibitor R3-GI.*

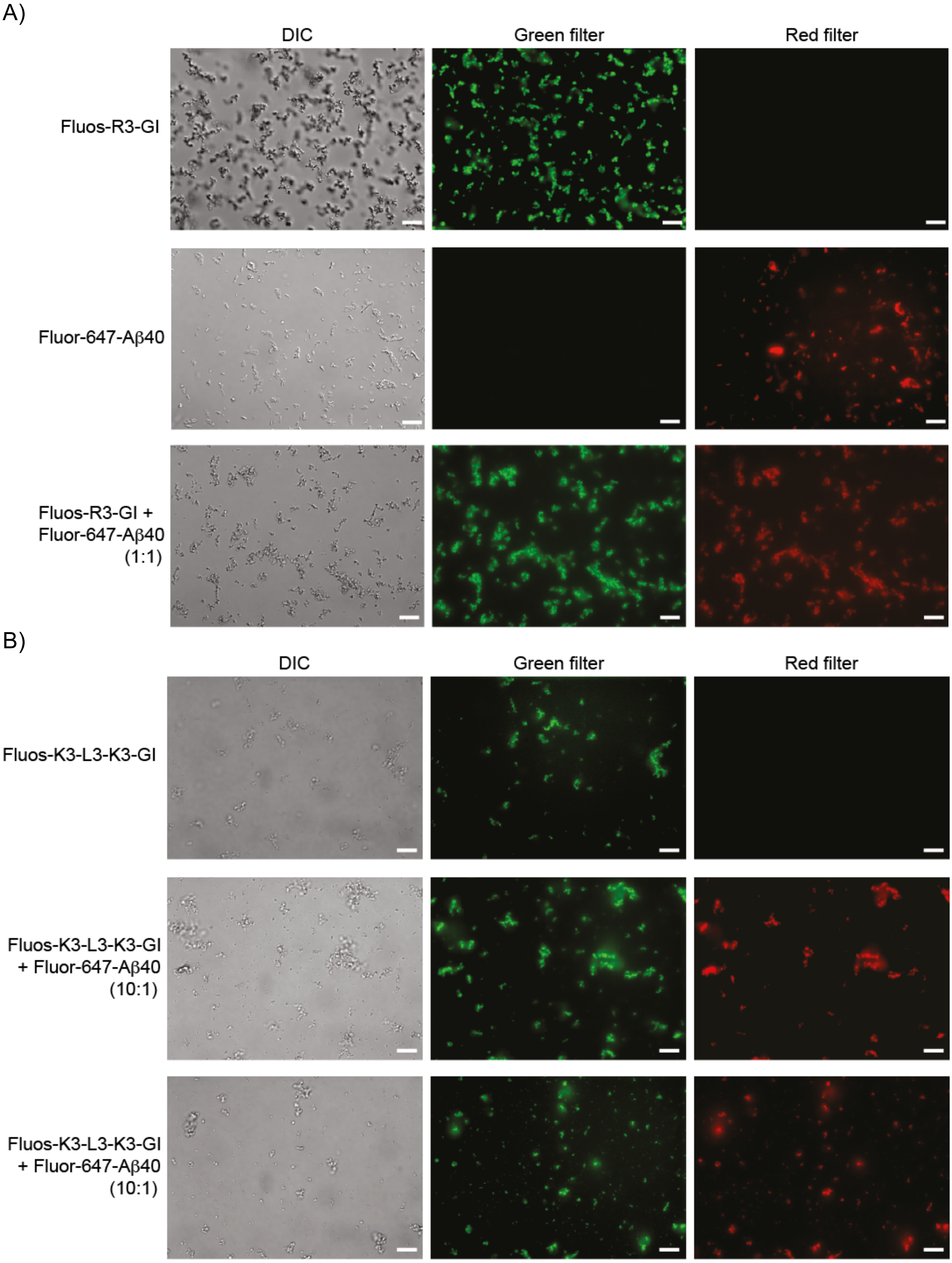

Supporting Figure 10. Colocalization of R3-GI and K3-L3-K3-GI with Aβ40. *(A) DIC and fluorescence microscopic images of Fluos-R3-GI (green) and Fluor-647 labeled Aβ40 (red). The top two control panels indicate (at the given threshold) molecular structures that originate only from either R3-GI or Aβ40, respectively. The bottom panels indicate co-localization of R3-GI and Aβ40. (B) DIC and fluorescence microscopic images of Fluos-labeled K3-L3-K3-GI (green) and Fluor-647 labeled Aβ40 (red). Similar as in figure (A), K3-L3-K3-GI and Aβ40 co-localize.*

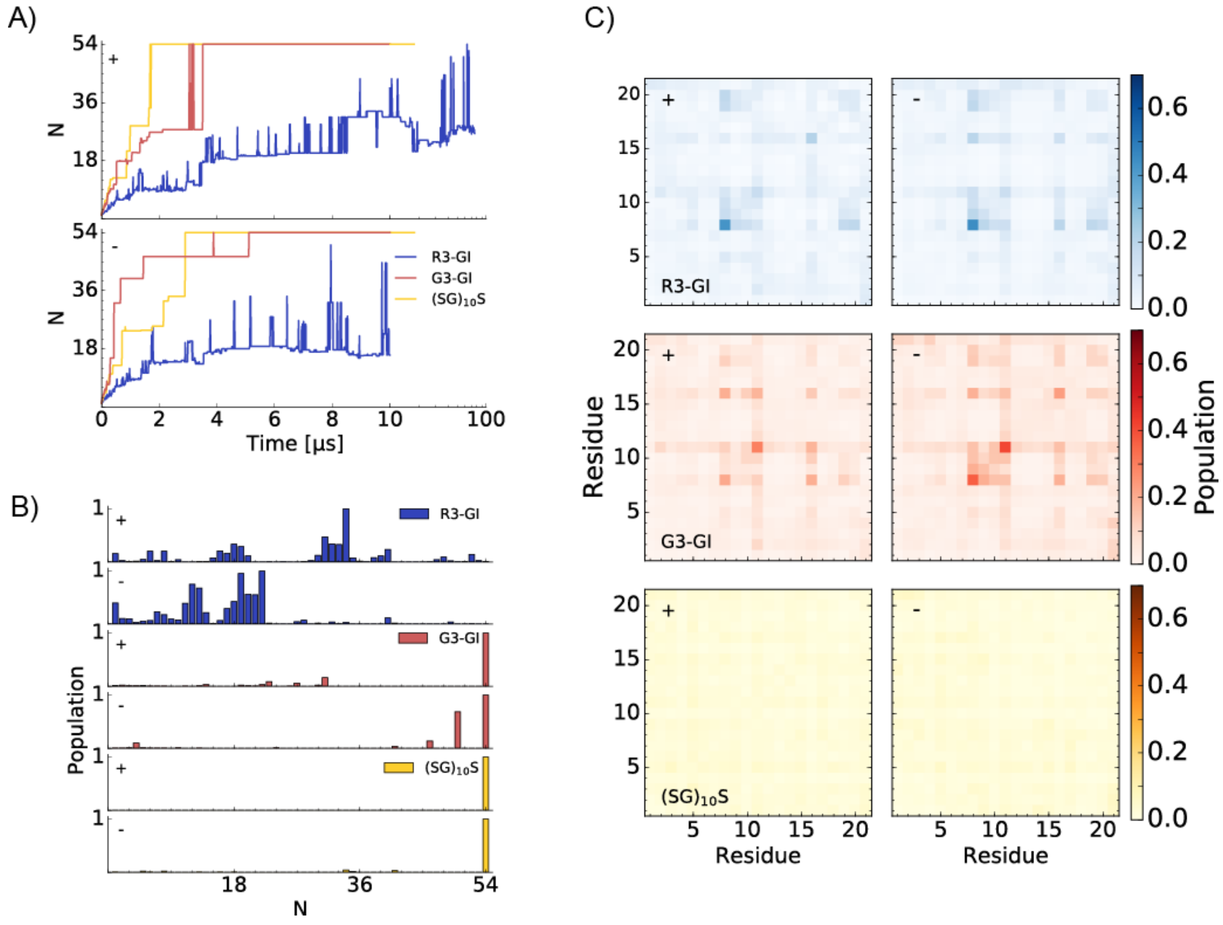

Supporting Figure 11. Coarse-grain MD simulations of R3-GI, G3-GI and (SG)$_{10}$S self-assembly. *A) Average oligomer size during the simulations with R3-GI, G3-GI and (SG)$_{10}$S with (+) and without (-) an elastic network. The simulation was performed using a total of 54 monomers. A value of N=54 would imply that a single oligomer is formed encompassing all the 54 simulated molecules; by contrast, a value of N=1 would indicate that all 54 molecules are monomeric in solution and no oligomers are formed. B) Distribution of the oligomer size over the simulations with (+) and without (-) an elastic network. C) Intermolecular contact probability map averaged over the simulations of the different constructs with (+) and without (-) an elastic network.*

Table S1. Determination of molecular weights of synthesized ISMs and analogs[a] by MALDI-TOF-MS.

Peptide Abbreviation	Peptide sequence	$[M+H]^+$ calc.[b]	$[M+H]^+$ found[b]
**R3-GI**	ATQRLANFLVH-**RRR**-NF(N-Me)GA(N-Me)ILS	2467.39	2467.86
**R3-GI***[c]	ATQRLANFLVH-**RRR**-NF(N-Me)GA(N-Me)ILS[b]	2512.39	2512.49
**G3-GI**	ATQRLANFLVH-**GGG**-NF(N-Me)GA(N-Me)ILS	2170.15	2170.96
**K3-L3-K3-GI**	*KKK*-ATQRLANFLVH-**LLL**-NF(N-Me)GA(N-Me)ILS-*KKK*	3107.89	3107.85
**R3-G**	ATQRLANFLVH-**RRR**-NF(N-Me)GAILS	2453.39	2453.82
**R3-I**	ATQRLANFLVH-**RRR**-NFGA(N-Me)ILS	2453.39	2453.91
**R3**	ATQRLANFLVH-**RRR**-NFGAILS	2440.36	2440.64

[a] C-terminal amides; [b] M, monoisotopic mass; [c] R3-GI analog containing ^{13}C-,^{15}N-labeled amino acids (red).

Table S2. Assignments for R3-GI, conformer 1 (solution-state NMR)

aa	H	Hα	Hβ	Hγ	Hδ	additional H	Cα	Cβ	Cγ	Cδ
A1	-	3.963	1.007	-	-	-	-	-	-	-
T2	7.979	3.772	-	0.873	-	-	-	-	-	-
Q3	8.423	3.966	1.637, 1.720	2.016	-	7.326, 6.650	-	-	-	-
R4	8.317	3.939	1.393, 1.446	1.228, 1.267	2.793	-	-	-	-	-
L5	8.163	3.991	1.228, 1.299	-	0.523, 0.583	-	51.956	39.268	24.051	20.338, 21.906
A6	8.114	3.853	0.961	-	-	-	49.721	16.154	-	-
N7	8.073	4.239	2.380	-	7.359, 6.622	-	-	-	-	-
F8	7.815	4.212	2.706, 2.794	-	-	6.995, 6.880	55.018	36.398	-	-
L9	7.808	3.913	1.095, 1.232	-	0.487, 0.545	-	52.181	39.276	23.792	20.468, 21.979
V10	7.705	3.600	1.631	0.494, 0.566	-	-	59.650	29.675	17.900	-
H11	8.080	4.255	2.679, 2.752	-	6.653	7.517	-	-	-	-
R12	8.273	3.918	1.378, 1.461	1.307	2.800	6.949	-	-	-	-
R13	8.273	3.920	1.430, 1.491	1.207, 1.253	2.810	6.910	-	-	-	-
R14	8.220	3.923	1.477, 1.401	-	2.809	6.911	-	-	-	-
N15	7.967	4.319	2.340, 2.388	-	6.644, 7.356	-	-	-	-	-
F16	7.968	4.319	2.616, 2.735	-	7.358	6.910	-	-	-	-
G17	2.647	3.664, 3.737	-	-	-	-	-	-	-	-
A18	7.986	4.371	0.954	-	-	-	-	-	-	-
I19	2.773	4.370	1.710	0.531, 0.644, 1.029	0.481	-	-	-	-	-
L20	8.233	4.052	1.227, 1.316	-	0.485, 0.531	-	51.917	38.927	23.989	19.915, 21.800
S21	8.045	4.051	3.491, 3.539	-	-	-	55.031	60.835	-	-

Table S3. Assignments for R3-GI, conformer 2 (solution-state NMR)

aa	H	Hα	Hβ	Hγ	Hδ	additional H
N7	8.054	-	-	-	-	-
F8	-	4.222	-	-	-	-
L9	7.841	3.952	1.101, 1.233	-	0.463, 0.521	-
V10	7.665	3.642	1.551	0.354, 0.468	-	-
H11	8.324	4.291	2.661, 2.795	-	6.679	7.491
N15	8.201	4.290	2.302, 2.357	-	7.347	-
F16	7.856	4.584	2.540, 2.661	-	-	6.994, 6.861
G17	2.544	3.751, 3.938	-	-	-	-
A18	8.274	4.384	0.969	-	-	-
I19	2.756	4.384	1.651	0.439	0.355	-
L20	8.130	4.296	-	-	0.499, 0.565	-
S21	8.129	4.050	3.456, 3.503	-	-	-

Table S4: Long-range NOE contacts for R3-GI, conformer 1. Only cross peaks involving residues that are more than 2 residues apart are included in the table. No prochiral assignments have been carried out for side chains. Suffixes *a* and *b* are therefore used to indicate these protons.

assignment F1 (atom 1)	assignment F2 (atom 1)	long NOE contacts
3GlnHa	6AlaH	
5LeuH	2ThrHg1	
5LeuHdb*	8PheHz	
5LeuHdb*	8PheHe*	
7AsnHa	10ValHgb*	
7AsnHa	10ValHga*	
7AsnHba	10ValHb	
7AsnHba	10ValHgb*	
7AsnHba	10ValHga*	
7AsnHd2a	10ValHga*	
7AsnHd2a	10ValHgb*	
7AsnHd2b	10ValHga*	
7AsnHd2b	10ValHgb*	
8PheH	5LeuHa	
8PheH	5LeuHbb	
8PheHe*	5LeuHbb	
8PheHe*	5LeuHa	
8PheHe*	5LeuHba	
8PheHz	5LeuHa	
8PheHz	5LeuHbb	
8PheHz	5LeuHba	
10ValH	7AsnHba	
10ValHb	7AsnHba	
10ValHga*	7AsnHd2a	
10ValHga*	7AsnHd2b	
10ValHga*	7AsnHba	
10ValHgb*	7AsnHd2b	
10ValHgb*	7AsnHd2a	
10ValHgb*	7AsnHba	
10ValHgb*	18AlaHb*	***
15AsnHba	18AlaHb*	
15AsnHbb	18AlaHb*	
16PheHd*	21SerHbb	***
16PheHd*	21SerHba	***
16PheHd*	20LeuHba	***
16PheHz	21SerHba	***
16PheHz	19IleHd1*	

16PheHz	21SerHbb	***
18AlaHb*	15AsnHba	
20LeuH	10ValHgb*	***

Table S5: Long-range NOE contacts for R3-GI, conformer 2. Only cross peaks involving residues that are more than 2 residues apart are included in the table. No prochiral assignments have been carried out for side chains. Suffixes *a* and *b* are therefore used to indicate these protons.

Assignment F1 (atom 2)	Assignment F2 (atom 2)	Long NOE contacts
7AsnH	19IleHg2*	***
16PheHe*	10ValHgb*	***
16PheHe*	19IleHd1*	
16PheHe*	19IleHg2*	
16PheHz	19IleHg2*	
16PheHz	19IleHd1*	

Table S6. Chemical shift assignments for K3-L3-K3-GI induced Aβ40 fibrils (MAS solid-state NMR)

aa	N	Nζ	C	Cα	Cβ	Cγ	Cδ	Cε
K16	124.59	67.74	172.63	53.88	33.71	24.21	27.57	40.34
L17	122.20	-	172.96	52.36	45.33	26.00	24.17	-
V18	119.40	-	171.18	58.62	33.56	19.10	-	-
F19	124.45	-	171.25	53.36	42.32	-	-	128.04
F20	125.13	-	170.63	54.22	41.39	136.31	-	129.73
A21	126.69	-	173.10	48.06	19.22	-	-	-
E22	118.10	-	173.83	51.71	32.15	34.62	181.24	-
D23	122.57	-	172.46	52.40	35.74	179.85	-	-
V24	121.46	-	173.26	58.17	33.36	18.31	-	-
G25	114.95	-	171.32	45.46	-	-	-	-
S26	110.82	-	173.03	55.56	65.32	-	-	-
N27	117.24	-	174.75	51.28	39.51	178.21	-	-
K28	115.78	67.71	172.30	53.04	34.22	23.79	28.33	40.32
G29	107.63	-	170.11	41.42	-	-	-	-
A30	126.55	-	173.40	48.10	21.74	-	-	-
I31	123.83	-	172.57	59.14	38.40	26.26, 17.47	11.70	-
I32	123.48	-	174.45	55.78	40.62	24.87, 15.32	12.07	-
G33	113.88	-	170.52	47.19	-	-	-	-
L34	113.40	-	171.93	51.74	44.24	26.24	23.97, 23.01	-
M35	122.73	-	171.90	52.27	32.96	30.59	-	19.52
V36	122.44	-	172.36	57.62	34.43	20.20	-	-
G37	110.66	-	169.76	42.61	-	-	-	-
G38	104.42	-	169.13	44.73	-	-	-	-
V39	117.69	-	171.75	58.17	34.29	18.62	-	-
V40	-	-	178.93	61.61	31.63	19.97	-	-

References

[1] E. Andreetto, L. M. Yan, M. Tatarek-Nossol, A. Velkova, R. Frank, A. Kapurniotu, Angew. Chem. Int. Ed. Engl. 2010, 49, 3081-3085.
[2] J. Hendrix, W. Schrimpf, M. Holler, D. C. Lamb, Biophys. J. 2013, 105, 848-861.
[3] W. Schrimpf, A. Barth, J. Hendrix, D. C. Lamb, Biophys. J. 2018, 114, 1518-1528.
[4] P. Kapusta, Picoquant Application Note 2010.
[5] C. A. Schneider, W. S. Rasband, K. W. Eliceiri, Nat. Methods 2012, 9, 671-675.
[6] W. F. Vranken, W. Boucher, T. J. Stevens, R. F. A. Pajon, M. Llinas, E. L. Ulrich, J. L. Markley, J. Ionides, E. D. Laue, Proteins 2005, 59, 687-696.
[7] a) A. Jerschow, N. Müller, J. Magn. Reson. 1997, 125, 372-375; b) A. Jerschow, N. Muller, J. Magn. Reson. 1998, 132, 13-18.
[8] J. M. Lopez del Amo, M. Schmidt, U. Fink, M. Dasari, M. Fändrich, B. Reif, Angewandte Chemie Int. Edt. Engl. 2012, 51, 6136 –6139.
[9] M. Bonomi, C. Camilloni, M. Vendruscolo, Sci. Rep. 2016, 6.
[10] D. Van der Spoel, E. Lindahl, B. Hess, G. Groenhof, A. E. Mark, H. J. C. Berendsen, J. Comput. Chem. 2005, 26, 1701-1718.
[11] G. A. Tribello, M. Bonomi, D. Branduardi, C. Camilloni, G. Bussi, Comp. Phys. Commun. 2014, 185, 604-613.
[12] M. Bonomi, C. Camilloni, Bioinformatics 2017, 33, 3999-4003.
[13] S. Piana, A. G. Donchev, P. Robustelli, D. E. Shaw, J. Phys. Chem. B 2015, 119, 5113-5123.
[14] S. J. Marrink, H. J. Risselada, S. Yefimov, D. P. Tieleman, A. H. de Vries, J. Phys. Chem. B 2007, 111, 7812-7824.
[15] E. Andreetto, E. Malideli, L.-M. Yan, M. Kracklauer, K. Farbiarz, M. Tatarek-Nossol, G. Rammes, E. Prade, T. Neumüller, A. Caporale, A. Spanopoulou, M. Bakou, B. Reif, A. Kapurniotu, Angew. Chem. Int. Ed. 2015, 54, 13095-13100.
[16] D. S. Wishart, Sykes, B.D., J. Biomol. NMR 1994, 4, 171-180.
[17] Y. Shen, F. Delaglio, G. Cornilescu, A. Bax, J. Biomol. NMR 2009, 44, 213-223.
